# CircPTPRA promotes the progression of pancreatic ductal adenocarcinoma via the miR‐140‐5p/LMNB1 axis

**DOI:** 10.1002/cam4.5869

**Published:** 2023-04-11

**Authors:** Wen Fu, Xianxing Wang, Jifeng Xiang, Shengkai Chen, Renpei Xia, Fuming Xie, Bojing Chi, Fanbo Qin, Zhuo Li, Li Mou, Chuanming Xie, Huaizhi Wang

**Affiliations:** ^1^ Chongqing Medical University Chongqing 400016 China; ^2^ Chongqing Institute of Green and Intelligent Technology, Chinese Academy of Sciences Chongqing 400174 China; ^3^ Chongqing School University of Chinese Academy of Sciences Chongqing 400013 China; ^4^ Department of Hepatopancreatobiliary Surgery Chongqing General Hospital Chongqing 401147 China; ^5^ Chinese Academy of Sciences Beijing 101400 China; ^6^ NHC Key Laboratory of Birth Defects and Reproductive Health Chongqing Population and Family Planning Science and Technology Research Institute Chongqing 400020 China; ^7^ Key Laboratory of Hepatobiliary and Pancreatic Surgery, Institute of Hepatobiliary Surgery Southwest Hospital, Third Military Medical University (Army Medical University) Chongqing 400038 China

**Keywords:** CircPTPRA, epithelial‐mesenchymal transition, LMNB1, MiR‐140‐5p, pancreatic ductal adenocarcinoma

## Abstract

**Background:**

Growing evidences suggest that circular RNAs (circRNAs) are important factors in cancer progression. Nevertheless, the role of circRNAs in the progression of pancreatic ductal adenocarcinoma (PDAC) remains unclear.

**Methods:**

CircPTPRA was identified based on our previous circRNA array data analysis. Wound healing, transwell, and EdU assays were performed to investigate the effect of circPTPRA on the migration, invasion, and proliferation of PDAC cells in vitro. RNA pull‐down, fluorescence in situ hybridization (FISH), RNA immunoprecipitation (RIP), and dual‐luciferase reporter assays were conducted to verify the binding of circPTPRA with miR‐140‐5p. Subcutaneous xenograft model was constructed for in vivo experiment.

**Results:**

CircPTPRA was significantly upregulated in PDAC tissues and cells compared to normal controls. Moreover, circPTPRA overexpression was positively correlated with lymph node invasion and worse prognosis in PDAC patients. In addition, overexpression of circPTPRA promoted PDAC migration, invasion, proliferation, and epithelial‐mesenchymal transition (EMT) in vitro and in vivo. Mechanistically, circPTPRA upregulates LaminB1 (LMNB1) expression by sponging miR‐140‐5p and ultimately promotes the progression of PDAC.

**Conclusions:**

This study revealed that circPTPRA plays an important role in the progression of PDAC by sponging miR‐140‐5p. It can be explored as a potential prognostic marker and therapeutic target for PDAC.

## Graphical abstract

Our study confirmed that circPTPRA is abnormally upregulated in PDAC and is closely associated with local lymph node invasion and a poor prognosis. More importantly, the activation of the circPTPRA/miR‐140‐5p/LMNB1 signaling pathway promoted PDAC migration, invasion, proliferation, and EMT in vitro and in vivo. However, the mechanism of circPTPRA activation needs further study, and the expression of circPTPRA should be further evaluated in blood, urine, and exosomes to improve its clinical application value.

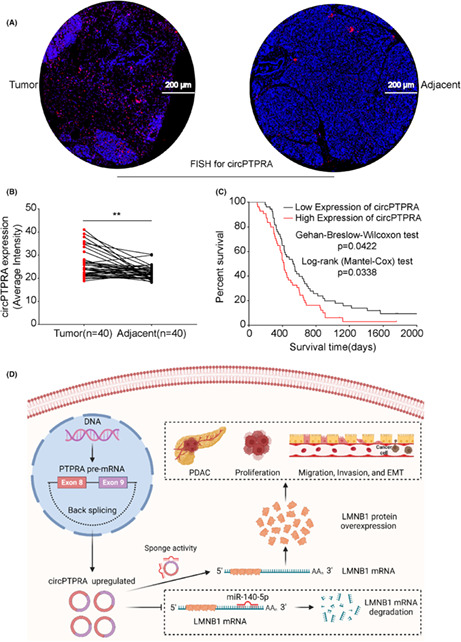

## 1 INTRODUCTION

The increasing morbidity and mortality rates of PDAC present a serious health burden worldwide.^1^, ^2^ According to the latest research from the United States, PDAC ranks 10th (males) or 8th (females) among cancers in terms of incidence, but ranks 4th in terms of mortality in both males and females.^3^ The early symptoms of PDAC are ambiguous, and local invasion or distant metastasis are prone to occur.^4^ Most patients miss the opportunity for surgery because they are already in the advanced stage of the disease when they are diagnosed.^5^ Compared with other malignant tumors, PDAC is not effectively treated with combined therapy, including immunotherapy and targeted therapy, and the 5‐year survival rate is still less than 10%.^2^, ^3^ Therefore, it is necessary to further explore the molecular network of PDAC and find more reliable biomarkers and potential therapeutic targets.

CircRNAs, as a special type of RNA, were first found in plant viruses.^6^ CircRNAs belong to the long‐chain noncoding RNA family and have a covalent closed‐loop structure produced by the back‐splicing of pre‐mRNA transcripts.^7^ CircRNAs lack 5′ caps and 3′ poly(A) tails, they can resistant to exonuclease and stably present in eukaryotic cells.^8^ The cellular roles of circRNAs include modulating transcription, regulating splicing, sponging microRNAs (miRNAs), forming circular RNA–protein complexes, and being translated into proteins or peptides.^8^, ^9^ In recent years, the roles of circRNAs in tumor have received much attention,^10^ especially certain circRNAs participate in the process of PDAC.^11^ For instance, the circEYA3/miR‐1294/c‐Myc axis promotes the progression of PDAC by inducing energy production,^12^ the circNEIL3 regulatory loop promotes PDAC progression via miRNA sponging and A‐to‐I RNA editing.^13^

MiRNAs are approximately 18–22 nucleotides long and have binding sequences that are partially complementary to the target mRNA transcripts, known as miRNA response elements (MREs).^14^, ^15^ As endogenous noncoding RNAs, miRNAs directly bind to the 3′UTR of mRNAs through MREs to form the RNA‐induced silencing complexes, which regulate the expression of target genes at the posttranscriptional level.^16^, ^17^ In addition to the traditional microRNA/RNA function, there is an opposite RNA/microRNA function known as the competing endogenous RNA (ceRNA) mechanism, which was first reported in 2011.^18^ CircRNAs, lncRNAs, and pseudogenes block the inhibitory effect of miRNAs on mRNAs by competitively binding to the MREs of miRNAs.^18^, ^19^ Here is a classic example, ciRS‐7 contains more than 70 selectively retained miRNA target sites and inhibits miR‐7 activity by binding with miR‐7 and Argonaute (AGO) protein, resulting in increased levels of miR‐7 targets.^20^ Therefore, the ceRNA mechanism regulated by circRNAs enriches the RNA interaction network and is a hot topic in the field of PDAC research.^21^, ^22^

In order to reveal the potential role of circRNAs in PDAC, we previously investigated the expression of circRNAs in 20 PDAC tissues and paired normal adjacent tissues (NATs). We found that the expression of circRNAs was significantly different between PDAC tissues and NATs, and circRNAs could act as miRNAs sponges to regulate gene expression.^23^ Next, we comprehensively analyzed our data (GSE79634) and the similar data (GSE69362) from other team^24^ to screen out the key circRNAs in PDAC. Finally, we found that circPTPRA (also known as hsa_circPTPRA_009 or hsa_circ_0006117) plays a key role in PDAC by promoting the migration, invasion, proliferation, and EMT of PDAC in vitro and in vivo. Mechanistically, circPTPRA sponges miR‐140‐5p to upregulate LMNB1 expression and promote PDAC progression. Clinically, circPTPRA is highly expressed in PDAC and is significantly associated with regional lymph node invasion and poor prognosis. In summary, we demonstrated that the circPTPRA/miR‐140‐5p/LMNB1 axis plays a key role in PDAC progression, and circPTPRA may serve as a potential biomarker and therapeutic target in PDAC.

## 2 MATERIALS AND METHODS

### 2.1 PDAC tissue specimens

Tissue microarrays (TMAs) were made by Shanghai Outdo Biotech Company. These PDAC patients were evaluated for clinical staging according to the American Joint Commission on Cancer 8th edition guidelines. None of the PDAC patients received chemotherapy or/and radiotherapy before surgery. All patients were followed up regularly until August 2021. Informed consent was obtained from all patients prior to obtaining tissue samples, and all procedures were approved by the Medical Ethics Committee of Chongqing People's Hospital (KY 2021‐039‐01).

### 2.2 CircRNA array data

To determine the abnormal expression of circRNAs in PDAC, we previously measured circRNAs expression in 20 PDAC tissues and paired NATs. These data were published in the NCBI GEO datasets on October 7, 2016 (GSE79634).^23^ At the same time, we analyzed the GEO dataset (GSE69362) which contains circRNAs information for 6 PDAC tissues and paired NATs.^24^ Fold change ≥2 and *p* values < 0.05 were set as thresholds to identify the significant differentially expressed circRNAs (DEcircRNAs).

### 2.3 Cell lines and cell culture

The human normal pancreatic ductal epithelial cell line (HPDE6‐C7) was purchased from Genio Biotechnology Co., Ltd. The human PDAC cell lines AsPC‐1, BxPC‐3, CFPAC‐1, PANC‐1, and SW1990 were purchased from Genechem Co., Ltd. These cells were cultured in complete growth medium (Gibco) containing 10% fetal bovine serum (HyClone) under humidified conditions with 95% air, 5% CO_2_ at 37°C according to the manufacturer's instruction.

### 2.4 Plasmid and short interfering RNAs (siRNAs) transfection and lentiviral infection

To construct the overexpression plasmids of circPTPRA, LMNB1, EIF4A3, and FUS, the synthetic human circPTPRA cDNA was cloned into the pLC5‐ciR vector by Geneseed Biotech Co., Ltd., the synthetic human LMNB1, EIF4A3, and FUS cDNAs were cloned into the pEX‐3 vector by GenePharma, and the empty plasmid was used as a control. The siRNA sequences of circPTPRA, LMNB1, EIF4A3, and FUS, as well as the mimics and inhibitor of miR‐140‐5p were designed and synthesized by RiboBio Co., Ltd. Lipofectamine^TM^ 3000 (Invitrogen) and Opti‐MEM^TM^ (Gibco) were used as transient transfection reagents according to the manufacturer's instruction. Total RNA was extracted 48 h after transfection. The sequences of siRNAs, sh‐RNAs, plasmids, mimics and inhibitors are shown in Additional file 1 Table S1.

The overexpression and knockdown lentiviruses of circPTPRA and LMNB1 were constructed by Genechem Co., Ltd. Lentiviral infection was performed according to the manufacturer's instruction. Stable transfected cells were selected by culturing in medium containing 5 μg/mL puromycin (Beyotime), and the expression of circPTPRA and LMNB1 was confirmed by quantitative real‐time PCR (qRT–PCR).

### 2.5 RNA extraction and qRT–PCR analysis

Total RNA was extracted from PDAC cells by using the Ultrapure RNA Kit (CWBio). One microgram of total RNA in a final volume of 20 μL was used for reverse transcription with PrimeScript RT reagent Kit with gDNA Eraser (Takara) and T100™ Thermal cycler (Bio‐Rad). qRT–PCR was performed on the CFX96 PCR system (Bio‐Rad) by using TB Green Premix Ex Taq II (Takara) to detect the expression of the relevant genes following the manufacturer's protocol. The human GAPDH gene was used as the control. All primer sequences used for the qRT–PCR assay are summarized in Additional file 1 Table S1.

### 2.6 RNase R and actinomycin D treatment

Total RNA was treated with Ribonuclease R (Geneseed). Ten micrograms of total RNA was obtained and divided into two parts (5 μg/group); the samples were incubated at 37°C for 30 min with or without Ribonuclease R (3 U/μg), and then the expression of circPTPRA and PTPRA mRNA was measured by qRT–PCR.

Actinomycin D (2 μg/mL, CST) was added to the complete medium and incubated with the PDAC cells. Then, total RNA was extracted after 0, 3, 6, 12, and 24 h, respectively. Finally, the expression of circPTPRA and PTPRA mRNA was measured by qRT–PCR.

### 2.7 Western blotting

Appropriate amounts of protein were electrophoresed on the SurePAGE™, Bis‐Tris, 10 × 8, 4–20% gel (GenScript).Then, the proteins were transferred to a PVDF membrane (Millipore), and after blocking in 5% skim milk for 1 h, the PVDF membrane was soaked in primary antibody at 4°C overnight. Primary antibodies against LMNB1 (1/1000, ab229025, Abcam), EIF4A3 (1/1000, ab180573, Abcam), FUS (1/1000, ab243880, Abcam), GAPDH (1/1000, #5174, CST), E‐Cadherin (1/1000, #14472, CST), N‐Cadherin (1/1000, #13116, CST), Snail (1/1000, #3879, CST), β‐Catenin (1/1000, #8480, CST), Vimentin (1/1000, #5741, CST). The next day, the PVDF membrane was incubated with the specific secondary antibody for 1 h at room temperature. Finally, protein bands were visually detected by the ChemiDoc™ XRS+ (Bio‐Rad) and ECL detection system (Bioground), and the GAPDH band was used as the control.

### 2.8 FISH

The Cy3‐labeled circPTPRA probe and FAM‐labeled miR‐140‐5p probe were designed and synthesized by GenePharma. The FISH experiment was carried out according to the instruction of the RNA FISH kit (GenePharma) and images were photographed by confocal microscopy (Leica Microsystems). All probe sequences used for the FISH assay are summarized in Additional file 1 Table S1.

### 2.9 CircRNA pull‐down assay

A biotin‐labeled circPTPRA probe was designed and synthesized by RiboBio Co., Ltd. The RNA pull‐down assay was performed according to the instruction of the Pierce™ Magnetic RNA–Protein Pull‐Down Kit (Thermo Scientific). The probe sequences used for the RNA pull‐down assay are summarized in Additional file 1 Table S1.

### 2.10 Dual‐luciferase reporter assay

The wild‐type and mutant‐type circPTPRA and LMNB1 fragments were constructed and cloned into the dual‐luciferase reporter vector by GenePharma. Dual‐luciferase reporter plasmids (WT or Mut‐circPTPRA/LMNB1) were cotransfected into PDAC cells with miR‐140‐5p mimics or miR‐140‐5p mimics NC, respectively. After 48 h, Firefly and Renilla luciferase activities were assayed according to the Dual‐Glo^®^ Luciferase Assay System (Promega).

### 2.11 Nude mouse subcutaneous tumor model

The animal experimental model was approved by the Medical Ethics Committee of Chongqing People's Hospital (KY 2021‐039‐01). Five nude mice (4–5 weeks, Ensiweier) in each group were used to establish the subcutaneous tumorigenesis model. The suspensions (0.1 mL) contained 5 × 10^6^ PDAC cells, then the suspensions and high concentration of Matrigel (Corning) were mixed 1:1. Next, the suspensions and Matrigel mixture were randomly injected into the axilla of the right upper limb of nude mice. When subcutaneous tumors formed, they were measured weekly with calipers. The tumor volume = (length × width^2^)/2. Nude mice were sacrificed 30 days after PDAC cells were inoculated. The volume and weight of the tumors were measured, and the tumors were fixed with 4% paraformaldehyde (Biosharp) for immunohistochemistry (IHC) and hematoxylin–eosin (HE) staining.

### 2.12 EdU

EdU (5‐ethynyl‐2′‐ deoxyuridine) is an analog of thymidine and is incorporated into newly synthesized DNA. Detection is based on a copper‐catalyzed click reaction between an alkyne on EdU and a small azide molecule labeled with a fluorescence dye, such as Alexa Fluor 594. Through this fast click reaction, the newly synthesized DNA is labeled with Alexa Fluor 594 that can be detected using an appropriate fluorescence detection instrument. The first step of EdU experiment was cell culture: we cultivated an appropriate amount of AsPC‐1 and PANC‐1 cells in 6‐well plates. After overnight incubation, we transfected cells with the corresponding nucleic acid sequences. After 48 h incubation, we re‐cultivated an appropriate amount of transfected cells in 24‐well plates (three replicates per sample). The second step of EdU experiment was EdU labeling: after overnight incubation, we used BeyoClick^TM^ EdU‐594 cell proliferation detection kit (C0078S, Beyotime) for EdU experiment. According to the instruction of the kit, we diluted the EdU stock (10 mM) with culture medium (10% fetal bovine serum) at a ratio of 1:500 to obtain the 2x EdU working solution (20 μM). Then, we added 100 μL EdU working solution (2x, 20 μM) preheated at 37°C to cell cultures at an equal volume to achieve a final concentration of EdU at 10 μM. AsPC‐1 and PANC‐1 cells were cultured under conditions for 4 h (The length of the incubation time is dependent on cell type, which is usually 10% of cell cycle duration). The other steps of the EdU experiment were fixation, washing, permeabilization, EdU detection, nuclear staining, and observation: after EdU labeling, we removed the culture medium and followed the instruction to complete the above experimental steps in turn. Finally, result analysis: we used Image J to count the number of blue cells (representing nuclear cells) and red cells (representing proliferating cells), respectively. The number of EdU‐positive cells represents the cell proliferation ability.

### 2.13 Statistical analysis

GraphPad Prism version 7.0 (GraphPad Software) and SPSS version 23.0 (SPSS) were used for the statistical analysis. In this study, the quantitative data was expressed as the mean ± standard deviation. For univariate analysis: if the data followed a normal distribution, the differences between the two samples were analyzed by Student's *t* test, while one‐way analysis of variance was used for multiple groups; otherwise, the nonparametric Mann–Whitney test was used. Two‐way analysis of variance was used for multivariate analysis. The Kaplan–Meier method was used for survival analysis, and the log‐rank test and the Gehan‐Breslow‐Wilcoxon test were used for the comparison of survival curves. According to test sensitivity, the log‐rank test was applied to assess the long‐term survival effect, and the Gehan‐Breslow‐Wilcoxon test was applied to assess the short‐term survival effect. We utilized Pearson's correlation analysis to examine the correlation between two variables. We used *p* < 0.05 as an indicator of statistical significance.

## 3 RESULTS

### 3.1 Identification and characterization of circPTPRA in PDAC cells

**FIGURE 1:**
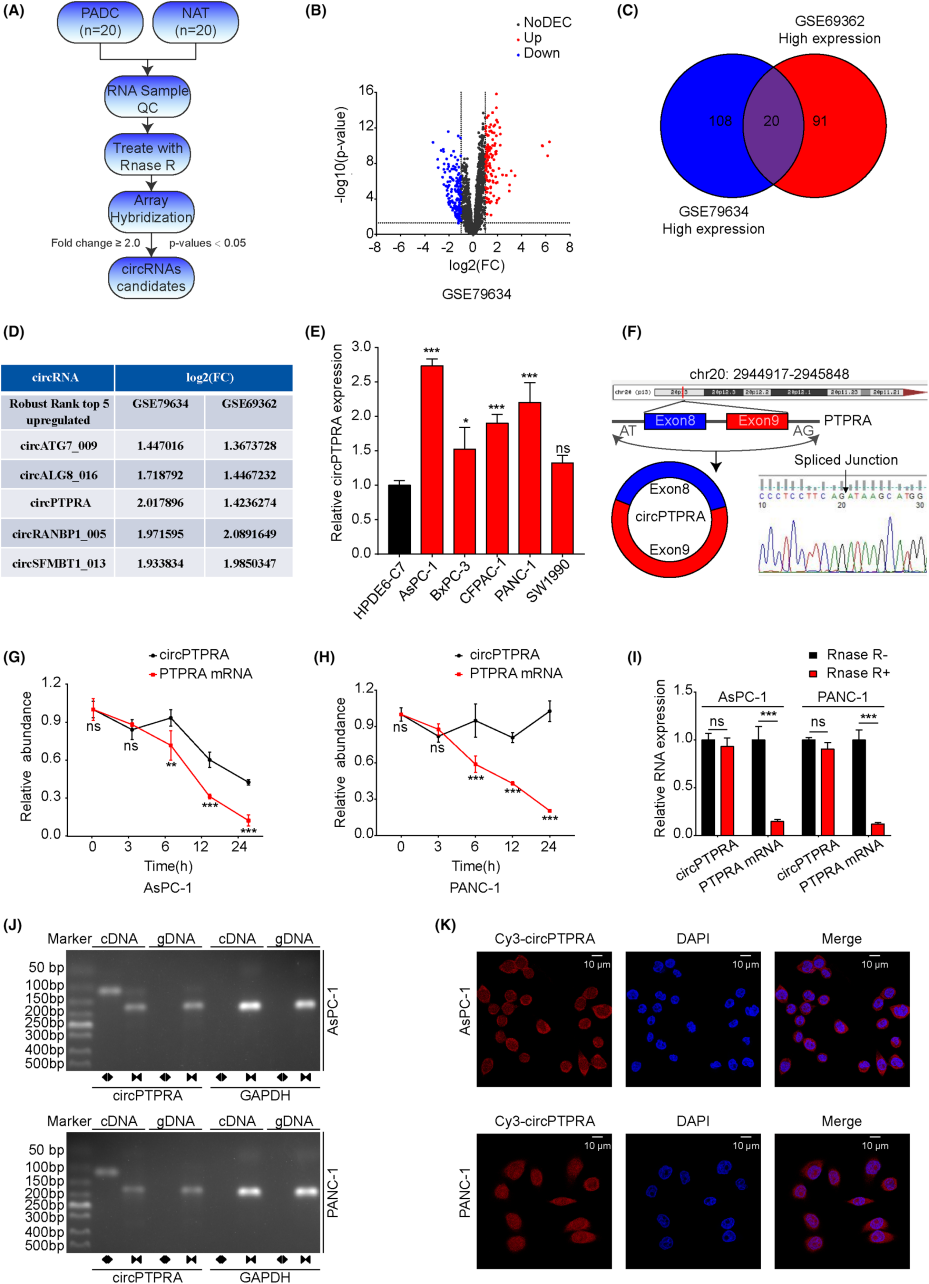
Identification and characterization of circPTPRA in PDAC cells. (A) The workflow for Arraystar Human CircRNA Array Analysis in 20 PDAC tissues and paired NATs. (B) Volcano plots showing 128 upregulated (red) and 161 downregulated (blue) circRNAs in PDAC tissues relative to paired NATs (fold change ≥2 and *p* values < 0.05). (C) Venn diagram showing 20 upregulated circRNAs in GSE79634 and GSE69362. (D) The top five upregulated circRNAs identified by RobustRankAggreg. (E) The expression of circPTPRA was determined by qRT–PCR in PDAC and HPDE cells. One‐way ANOVA was used. (F) Schematic illustration of the genomic loci and back splicing of circPTPRA. Sanger sequencing verified the spliced junction site of circPTPRA. (G, H) Actinomycin D treatment was used to evaluate the stability of circPTPRA and PTPRA mRNA in AsPC‐1 and PANC‐1 cells. Two‐way ANOVA was used. (I) The expression of circPTPRA and PTPRA mRNA was measured by qRT–PCR after RNase R (+) treatment in AsPC‐1 and PANC‐1 cells. Two‐way ANOVA was used. (J) Nucleic acid electrophoresis experiment was performed to detect the existence of circPTPRA and PTPRA mRNA in cDNA and genomic DNA (gDNA) samples from AsPC‐1 and PANC‐1 cells by using divergent and convergent primers. GAPDH was used as the negative control. (K) FISH assay showing the cellular localization of circPTPRA in AsPC‐1 and PANC‐1 cells. The circPTPRA probe was labeled with Cy3 (red), nuclei were stained with DAPI (blue). The FISH images were photographed at 630× magnification. Scale bar = 10 μm. The above data are presented as the mean ± SD of three independent experiments. *p < 0.05, **p < 0.01, ***p < 0.001.

Exploring the expression profile of circRNAs in PDAC can provide more reliable biomarkers and new therapeutic targets. Our team previously measured circRNAs expression in 20 PDAC tissues and NATs^23^ (Figure 1A). We found 289 DEcircRNAs (fold change ≥2 and *p* values < 0.05), of which 128 circRNAs were significantly upregulated and 161 circRNAs were significantly downregulated in PDAC tissues (Figure 1B). We also analyzed the data of GSE6936224^24^ and found that there were 111 significantly upregulated circRNAs in PDAC tissues (fold change ≥2 and *p* values < 0.05). Combined with the data of GSE79634 and GSE69362, we identified 20 highly expressed candidate circRNAs (Figure 1C). Next, differentially expressed circRNAs from the combined data of GSE79634 and GSE69362 were integrated and ranked by the RobustRankAggreg package. The top five upregulated circRNAs with *p* values < 0.05 were selected (Figure 1D). Among them, circPTPRA had the highest expression abundance and highly expressed in PDAC cells compared with HPDE6‐C7 cells (Figure 1E). Therefore, circPTPRA was chosen for further study. Through the annotations of circBase (http://www.circbase.org/)^25^ and circBank (http://www.circbank.cn/),^26^ we know that circPTPRA is formed by the circularization of exons 8 and 9 of the PTPRA gene (chr20: 2,944,917–2,945,848 strand: +) with a length of 421 bp. Sanger sequencing verified the back‐splicing site of circPTPRA (Figure 1F). Actinomycin D and RNase R treatment showed that compared with PTPRA mRNA, circPTPRA was more stable and had a longer half‐life (Figure 1G–I). Moreover, circPTPRA could be amplified by divergent primers from cDNA but not from gDNA in AsPC‐1 and PANC‐1 cells, respectively (Figure 1J). In addition, FISH experiment showed that Cy3‐labeled circPTPRA was mainly enriched in the cytoplasm of AsPC‐1 and PANC‐1 cells (Figure 1K). Taken together, these results confirmed that circPTPRA was significantly upregulated in PDAC cells and stably enriched in cytoplasm.

### 3.2 CircPTPRA promotes the progression of PDAC in vitro and in vivo

**FIGURE 2:**
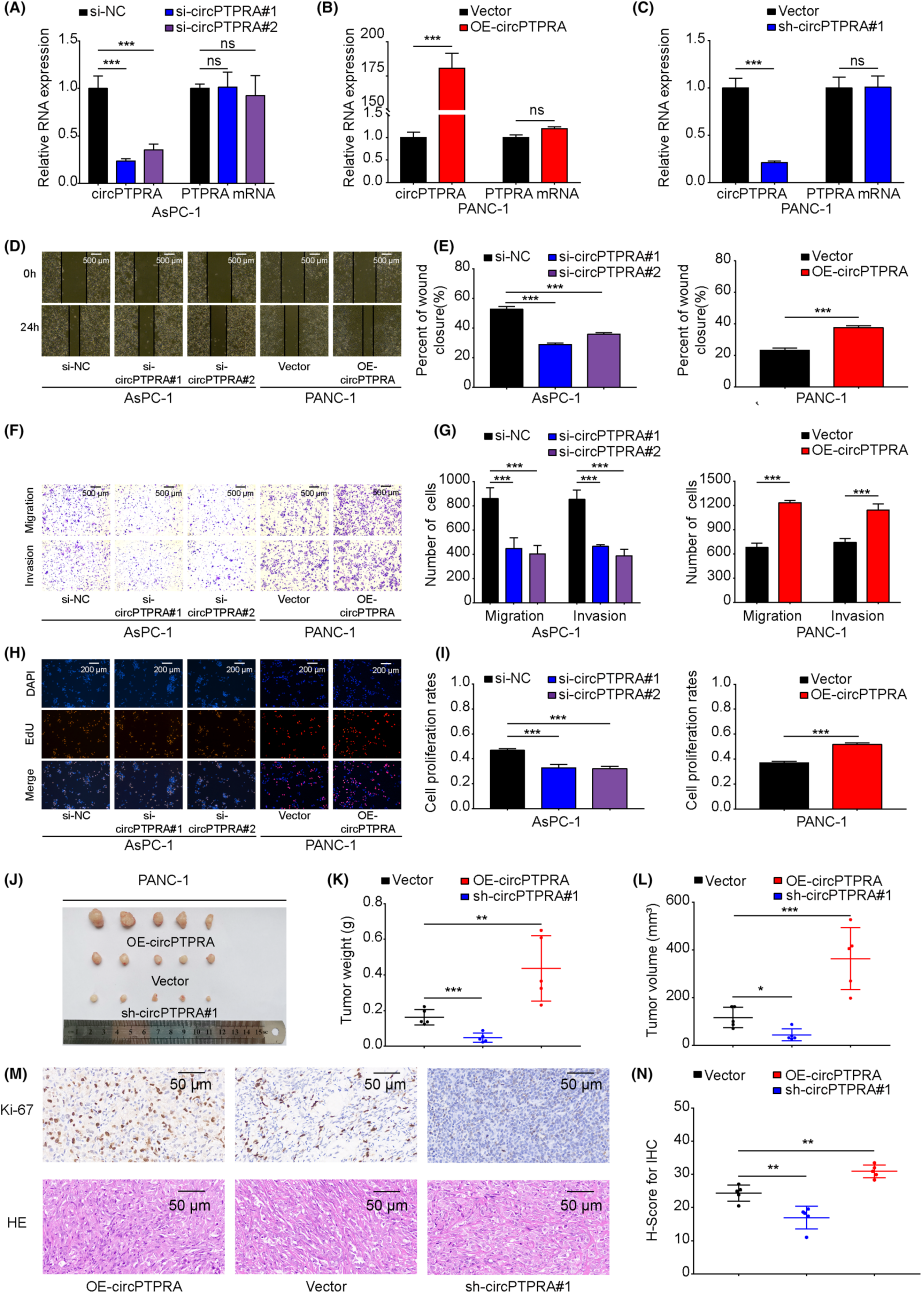
CircPTPRA promotes the progression of PDAC in vitro and in vivo. (A) The expression of circPTPRA and PTPRA mRNA was measured by qRT–PCR in AsPC‐1 cells transfected with specific siRNAs (si‐circPTPRA#1, si‐circPTPRA#2). Two‐way ANOVA was used. (B, C) The expression of circPTPRA and PTPRA mRNA was measured by qRT–PCR in PANC‐1 cells transfected with the OE‐circPTPRA and sh‐circPTPRA#1 plasmid. (D, E) Wound healing assay was used to evaluate the effect of circPTPRA on the migration ability of PDAC cells (AsPC‐1 cells transfected with si‐circPTPRA#1 and si‐circPTPRA#2, while PANC‐1 cells transfected with the OE‐circPTPRA plasmid). One‐way ANOVA and unpaired t test were used. The wound healing images were photographed at 40× magnification. Scale bar = 500 μm. (F, G) Transwell assay was used to evaluate the effect of circPTPRA on the migration and invasion ability of PDAC cells. Two‐way ANOVA was used. The transwell images were photographed at 40× magnification. Scale bar = 500 μm. (H, I) EdU assay was used to evaluate the effect of circPTPRA on the proliferation ability of PDAC cells. One‐way ANOVA and unpaired t test were used. The EdU images were photographed at 200× magnification. Scale bar = 200 μm. (J) Representative picture of subcutaneous xenograft tumors (*n* = 5 for each group). (K‐L) Tumor weight (g) and tumor volume (mm^3^) were analyzed. One‐way ANOVA was used. (M) IHC staining was used to evaluate Ki‐67 protein level in subcutaneous xenograft tumor (above), HE staining of subcutaneous xenograft tumor (below). The images of IHC staining and HE staining were photographed at 400× magnification. Scale bar = 50 μm. (N) H‐Score for IHC was analyzed. One‐way ANOVA was used. The above data are presented as the mean ± SD of three independent experiments. *p < 0.05, **p < 0.01, ***p < 0.001.

To verify the function of circPTPRA, we designed two specific siRNAs targeting the back‐splicing site of circPTPRA, named si‐circPTPRA#1 and si‐circPTPRA#2. We also constructed the overexpression and short hairpin RNA plasmid of circPTPRA (OE‐circPTPRA, sh‐circPTPRA#1). The expression of PTPRA mRNA was not affected after transfection with si‐circPTPRA, sh‐circPTPRA#1 or OE‐circPTPRA in AsPC‐1 and PANC‐1 cells, respectively (Figure 2A–C). Wound healing and transwell results showed that downregulation of circPTPRA in AsPC‐1 cells could significantly inhibit the migration and invasion ability of cells, whereas overexpression of circPTPRA in PANC‐1 cells could significantly promote the migration and invasion ability of cells (Figure 2D–G). EdU assay showed that the downregulation of circPTPRA in AsPC‐1 cells inhibited proliferation, while overexpression of circPTPRA in PANC‐1 cells promoted proliferation (Figure 2H,I). In nude mice, overexpression of circPTPRA promoted the growth of subcutaneous tumors (Figure 2J–L). IHC results showed that overexpression of circPTPRA induced the expression of Ki‐67 (Figure 2M,N). In summary, these results confirmed the role of circPTPRA in promoting the progression of PDAC.

### 3.3 CircPTPRA sponges miR‐140‐5p in PDAC cells

**FIGURE 3:**
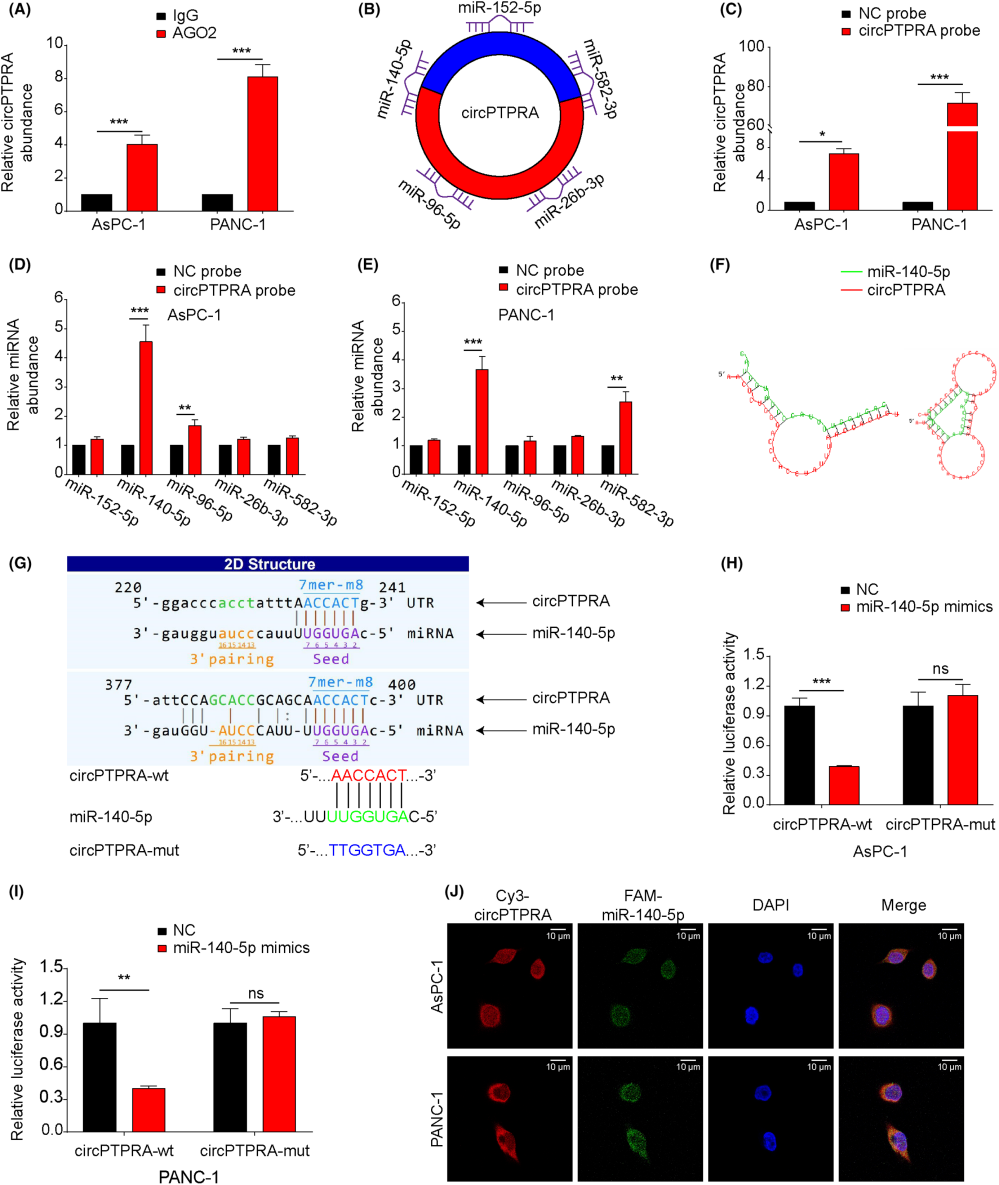
CircPTPRA sponges miR‐140‐5p in PDAC cells. (A) Anti‐AGO2 RIP and qRT–PCR were performed to detect the relative expression of circPTPRA. Anti‐IgG served as the negative control. Two‐way ANOVA was used. (B) Schematic illustration exhibiting five circPTPRA potential target miRNAs predicted by the TargetScan and miRanda databases. (C) The specificity of the biotin‐labeled circPTPRA probe was validated by the RNA pull‐down assay in AsPC‐1 and PANC‐1 cells. The random sequence NC probe served as the negative control. Two‐way ANOVA was used. (D, E) RNA pull‐down assay was used to detect the relative expression of five circPTPRA potential target miRNAs in AsPC‐1 and PANC‐1 cells. Two‐way ANOVA was used. (F) Schematic illustration showing the 3D structure of the potential binding sites between circPTPRA (red) and miR‐140‐5p (green). (G) Schematic illustration of the circPTPRA‐WT and circPTPRA‐Mut dual‐luciferase reporter vectors. (H, I) The relative intensity of firefly luciferase was detected after cotransfection with circPTPRA‐WT + miR‐140‐5p mimics/NC or circPTPRA‐Mut + miR‐140‐5p mimics/NC in AsPC‐1 and PANC‐1 cells, respectively. Two‐way ANOVA was used. (J) The colocalization of circPTPRA and miR‐140‐5p in AsPC‐1 and PANC‐1 cells was detected by the FISH assay. The circPTPRA probe was labeled with Cy3 (red), nuclei were stained with DAPI (blue), miR‐140‐5p probe was labeled with FAM (green). The FISH images were photographed at 1000× magnification. Scale bar = 10 μm. The above data are presented as the mean ± SD of three independent experiments. *p < 0.05, **p < 0.01, ***p < 0.001.

It has been reported that the regulation of downstream gene expression by sponging miRNAs is one of the main functions of circRNAs.^18^ The RIP results showed that circPTPRA could be significantly enriched by anti‐AGO2 (Figure 3A), which suggested that circPTPRA may have a ceRNA mechanism. The circRNA‐microRNA interaction was predicted with Arraystar's home‐made miRNA target prediction software based on TargetScan (https://www.targetscan.org/vert_80/) and miRanda (http://www.microrna.org/microrna/home.do).^27^, ^28^ We found that five miRNAs (miR‐140‐5p, miR‐152‐5p, miR‐582‐3p, miR‐26b‐3p, miR‐96‐5p) may directly bind to circPTPRA (Figure 3B). To verify the binding of the above five miRNAs and circPTPRA, we designed a biotin‐labeled circPTPRA probe for the RNA pull‐down assay. The results showed that circPTPRA was mainly captured by the biotin probe in AsPC‐1 and PANC‐1 cells compared with the random sequence NC probe (Figure 3C). Moreover, among the five potential target miRNAs, only miR‐140‐5p was significantly enriched by the biotin‐labeled circPTPRA probe in both AsPC‐1 and PANC‐1 cells (Figure 3D,E). The RNAhybrid database (https://bibiserv.cebitec.uni‐bielefeld.de/rnahybrid/)^29^ was used to predict the binding sequences between circPTPRA and miR‐140‐5p. The 3D structure showed that there were two binding sites between circPTPRA and miR‐140‐5p (Figure 3F). Then, we mutated the predicted binding sites of circPTPRA, and the wild‐type and mutant‐type circPTPRA fragments were constructed and cloned into the dual‐luciferase reporter vector, respectively (Figure 3G). In AsPC‐1 and PANC‐1 cells, the relative intensity of firefly luciferase was significantly decreased after cotransfection with wild‐type circPTPRA vector and miR‐140‐5p mimics compared to miR‐140‐5p mimics NC. However, when co‐transfected with mutant‐type circPTPRA vector, miR‐140‐5p mimics or miR‐140‐5p mimics NC did not affect the relative intensity of firefly luciferase (Figure 3H,I). The results indicated that circPTPRA could specifically bind miR‐140‐5p in the “seed” region. FISH experiment showed that Cy3‐labeled circPTPRA and FAM‐labeled miR‐140‐5p were colocalized in the cytoplasm (Figure 3J). Collectively, these data demonstrated that circPTPRA could directly sponge miR‐140‐5p.

### 3.4 CircPTPRA promotes PDAC progression by sponging miR‐140‐5p

**FIGURE 4:**
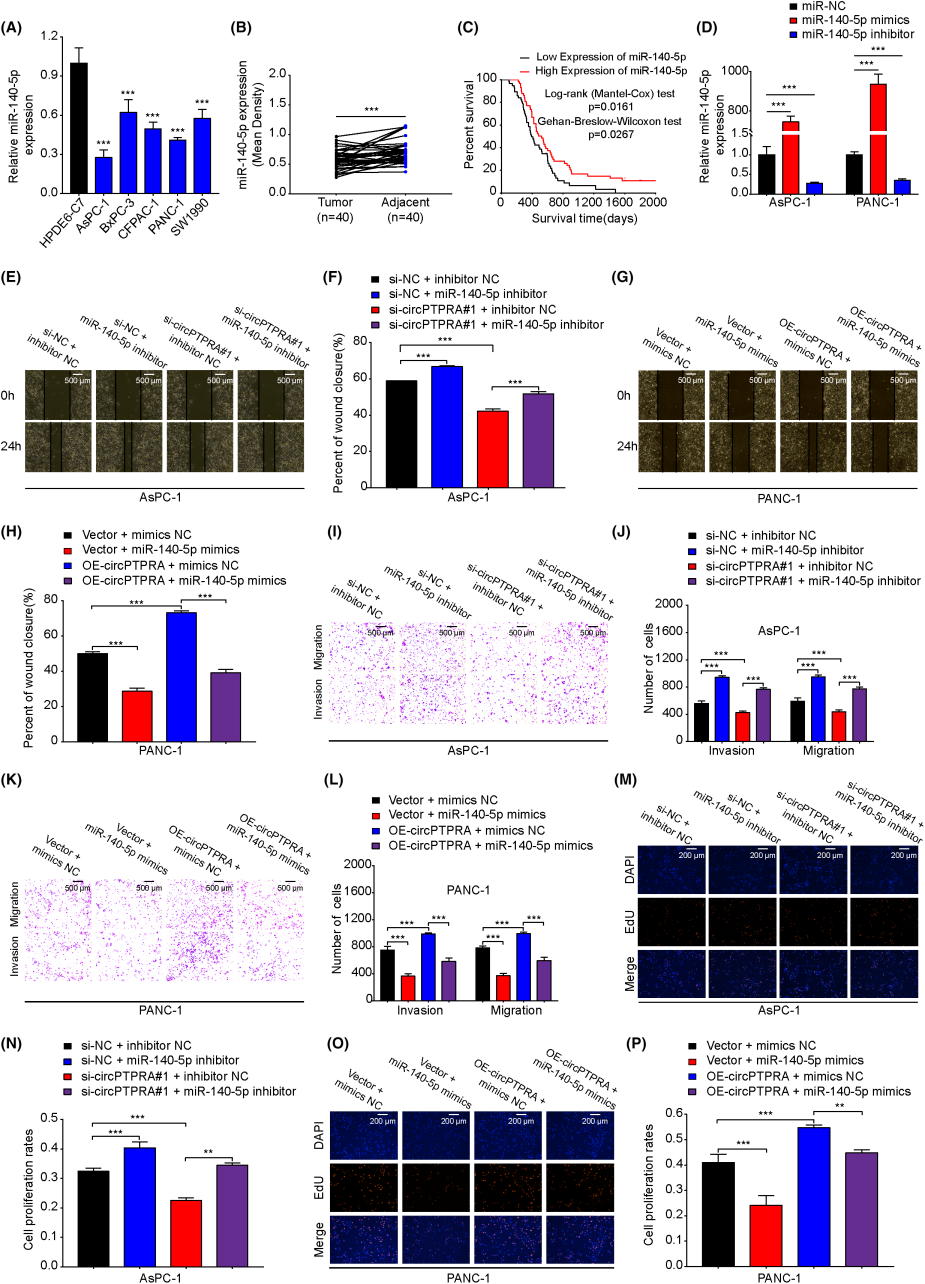
CircPTPRA promotes PDAC progression by sponging miR‐140‐5p. (A) The expression of miR‐140‐5p was determined by qRT–PCR in PDAC and HPDE cells. One‐way ANOVA was used. (B) The expression of miR‐140‐5p was determined by the miRNA scope assay in PDAC tissues (*n* = 40) and paired NATs (*n* = 40). The Mann–Whitney test was used. (C) Kaplan–Meier survival curves was used to analyze the overall survival of PDAC patients with high versus low miR‐140‐5p expression. (the median miR‐140‐5p expression was used as the cutoff value). (D) qRT–PCR was used to detect the efficiency of miR‐140‐5p inhibitor and mimics in AsPC‐1 and PANC‐1 cells, respectively. Two‐way ANOVA was used. (E–P) AsPC‐1 and PANC‐1 cells were divided into four groups for rescue experiments. The migration, invasion and proliferation capabilities of circPTPRA and miR‐140‐5p in AsPC‐1 and PANC‐1 cells were evaluated by wound healing (E–H), transwell (I–L) and EdU (M–P) assays. The images of wound healing and transwell assays were photographed at 40× magnification. Scale bar = 500 μm, the images of EdU were photographed at 200× magnification. Scale bar = 200 μm. One‐way and two‐way ANOVA were used. The above data are presented as the mean ± SD of three independent experiments. *p < 0.05, **p < 0.01, ***p < 0.001.

According to the literature, miR‐140‐5p acts as a tumor suppressor gene to inhibit PDAC progression.^30^ In this article, we found that the expression of miR‐140‐5p was decreased in PDAC cells (Figure 4A). Furthermore, we used the RNA scope assay to detect the expression of miR‐140‐5p in PDAC tissues. The results showed that miR‐140‐5p was significantly decreased in PDAC, which was positively correlated with the prognosis of PDAC patients (Figure 4B,C). To demonstrate whether circPTPRA promoted PDAC progression by sponging miR‐140‐5p, we performed rescue experiments in vitro. First, we used qRT–PCR to measure the efficiency of miR‐140‐5p mimics and inhibitor in AsPC‐1 and PANC‐1 cells, respectively (Figure 4D). The wound healing, transwell and EdU results showed that interfering with miR‐140‐5p in AsPC‐1 cells significantly promoted cell migration, invasion, proliferation, and reversed the anti‐tumor effects of si‐circPTPRA#1 (Figure 4E,F,I,J,M,N). On the other hand, upregulation of miR‐140‐5p significantly inhibited cell migration, invasion, proliferation, and reversed the pro‐tumor effects of OE‐circPTPRA in PANC‐1 cells (Figure 4G,H,K,L,O,P). Overall, these data suggested that miR‐140‐5p could act as a downstream target gene for circPTPRA and exert anti‐tumor effects in PDAC cells.

### 3.5 CircPTPRA promotes LMNB1 expression by competitively sponging miR‐140‐5p

**FIGURE 5:**
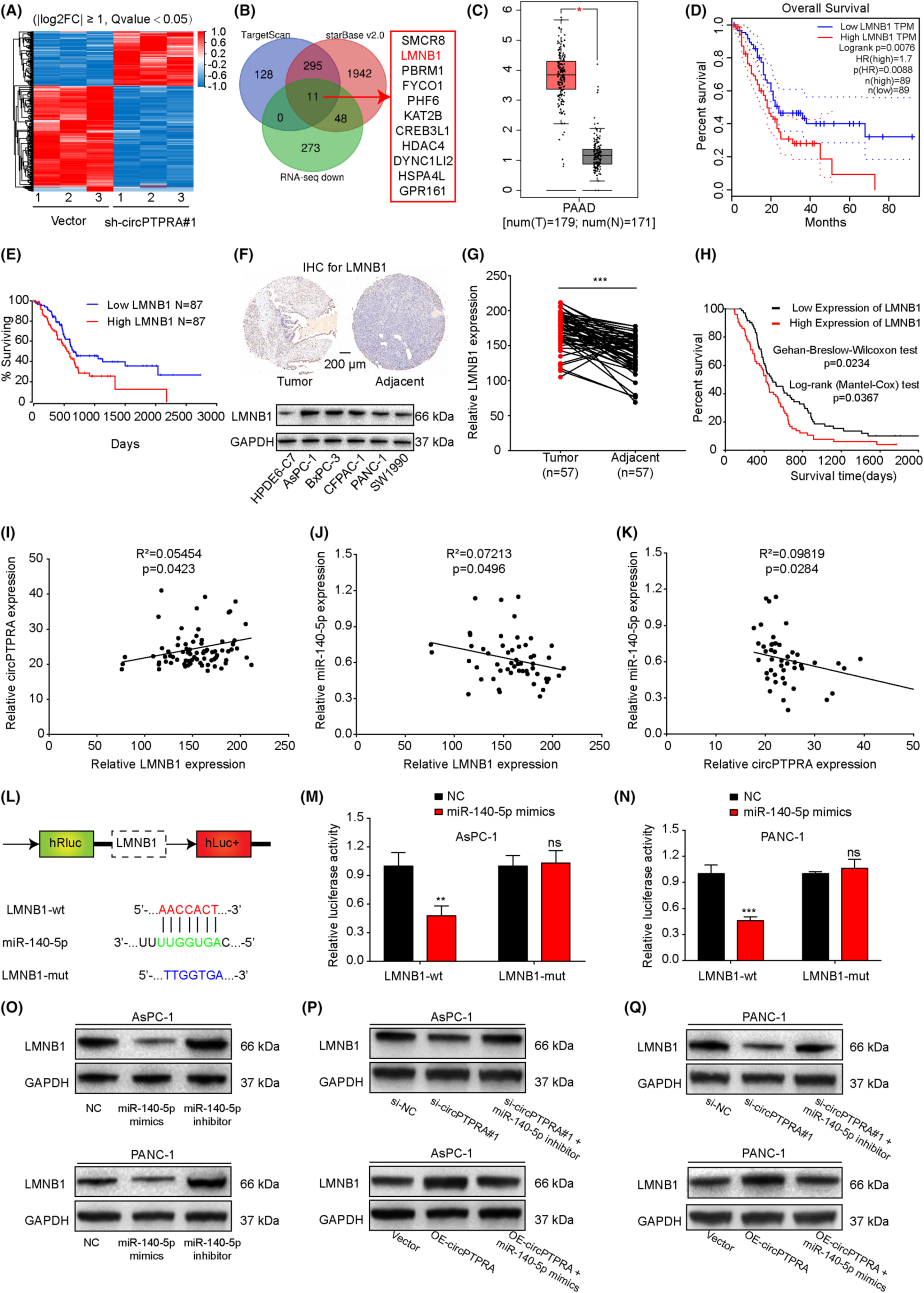
CircPTPRA promotes LMNB1 expression by competitively sponging miR‐140‐5p. (A) Heatmap showing the differentially expressed mRNAs in three sh‐circPTPRA#1 and three corresponding vector samples. Red and blue represented highly expressed and weakly expressed mRNAs, respectively (fold change ≥2 and *Q*‐values <0.05). (B) Venn diagram showing 11 miR‐140‐5p potential downstream target genes predicted by TargetScan, starBase v2.0 database and low expression data in RNA‐seq. (C) Data from GEPIA showing the expression of LMNB1 in pancreatic adenocarcinoma (PAAD) tissues (*n* = 179) and NATs (*n* = 171). (D) Data from GEPIA showing Kaplan–Meier survival curves of the overall survival of PAAD patients with high versus low LMNB1 expression. (E) Data from OncoLnc showing the Kaplan–Meier survival curves of the overall survival of PAAD patients with high versus low LMNB1 expression. (F) IHC staining of LMNB1 in PDAC tissues and paired NATs. The images of IHC were photographed at 50× magnification. Scale bar = 200 μm (above). The relative expression of LMNB1 in PDAC and HPDE cells was determined by western blotting (below). (G) The expression of LMNB1 in PDAC tissues (*n* = 57) and paired NATs (*n* = 57). The Mann–Whitney test was used. (H) Kaplan–Meier survival curves was used to analyze the overall survival of PDAC patients with high versus low LMNB1 expression. (the median LMNB1 expression was used as the cutoff value). (I–K) Linear regression analysis of the correlation between circPTPRA, miR‐140‐5p and LMNB1 expression in PDAC tissues and paired NATs. (L) Schematic illustration of the LMNB1‐WT and LMNB1‐Mut dual‐luciferase reporter vectors. (M‐N) The relative intensity of firefly luciferase was detected after cotransfection with LMNB1‐WT + miR‐140‐5p mimics/NC or LMNB1‐Mut + miR‐140‐5p mimics/NC in AsPC‐1 and PANC‐1 cells. Two‐way ANOVA was used. (O‐Q) After transfection with miR‐140‐5p mimics or inhibitor, si‐circPTPRA#1 or OE‐circPTPRA plasmid, and cotransfection with si‐circPTPRA#1 + miR‐140‐5p inhibitor or OE‐circPTPRA plasmid + miR‐140‐5p mimics in AsPC‐1 and PANC‐1 cells, respectively, the protein level of LMNB1 was detected by western blotting. The above data are presented as the mean ± SD of three independent experiments. *p < 0.05, **p < 0.01, ***p < 0.001.

To search potential downstream target genes of circPTPRA, we used stable sh‐circPTPRA#1 transfected PANC‐1 cells for whole transcriptome sequencing. With the fold change ≥2 and *Q*‐value <0.05, 332 genes were significantly downregulated, while 173 genes were significantly upregulated in the experimental group compared with the vector group (Figure 5A). MiRNAs usually interact with the 3′UTR of target genes and downregulate their expression. We combined the downregulated genes from RNA‐seq with data from TargetScan and starBase v2.0 (https://starbase.sysu.edu.cn/index.php)^27^, ^31^ to screen out 11 candidate genes which may be the potential downstream targets of circPTPRA and miR‐140‐5p (Figure 5B). Subsequently, the above 11 candidate genes were analyzed by the GEPIA (http://gepia.cancer‐pku.cn/) and OncoLnc (http://www.oncolnc.org/) databases. The results showed that only LMNB1 was abnormally highly expressed in PDAC and associated with a poor prognosis in patients (Figure 5C–E). Then, the expression of LMNB1 was detected in our clinical specimens, and the same results were obtained (Figure 5F–H). In addition, the expression of LMNB1 was positively correlated with the expression of circPTPRA, and the expression of LMNB1 and circPTPRA was negatively correlated with the expression of miR‐140‐5p in PDAC (Figure 5I–K). The results from the RNAhybrid database^29^ showed that LMNB1 shared the same sequence and bound the “seed” region of miR‐140‐5p, and then we constructed wild‐type and mutant‐type dual luciferase reporter plasmids targeting the 3′UTR of LMNB1 (Figure 5L). In AsPC‐1 and PANC‐1 cells, miR‐140‐5p mimics significantly reduced the relative activity of firefly luciferase in the LMNB1 wild‐type group, but the relative activity of firefly luciferase did not change significantly after transfection with miR‐140‐5p mimics in the LMNB1 mutant‐type group (Figure 5M,N). Mechanistically, the western blotting results showed that miR‐140‐5p mimics could significantly inhibit LMNB1 expression, while the miR‐140‐5p inhibitor could enhance LMNB1 expression (Figure 5O). On the other hand, the expression of LMNB1 was positively regulated by circPTPRA, but miR‐140‐5p mimics blocked the effect of OE‐circPTPRA on promoting LMNB1 expression, while the miR‐140‐5p inhibitor reversed the inhibition of LMNB1 expression induced by si‐circPTPRA#1 (Figure 5P,Q). In short, these results suggested that circPTPRA could competitively sponge miR‐140‐5p to promote LMNB1 expression.

### 3.6 The circPTPRA/miR‐140‐5p/LMNB1 axis promotes PDAC progression in vitro

**FIGURE 6:**
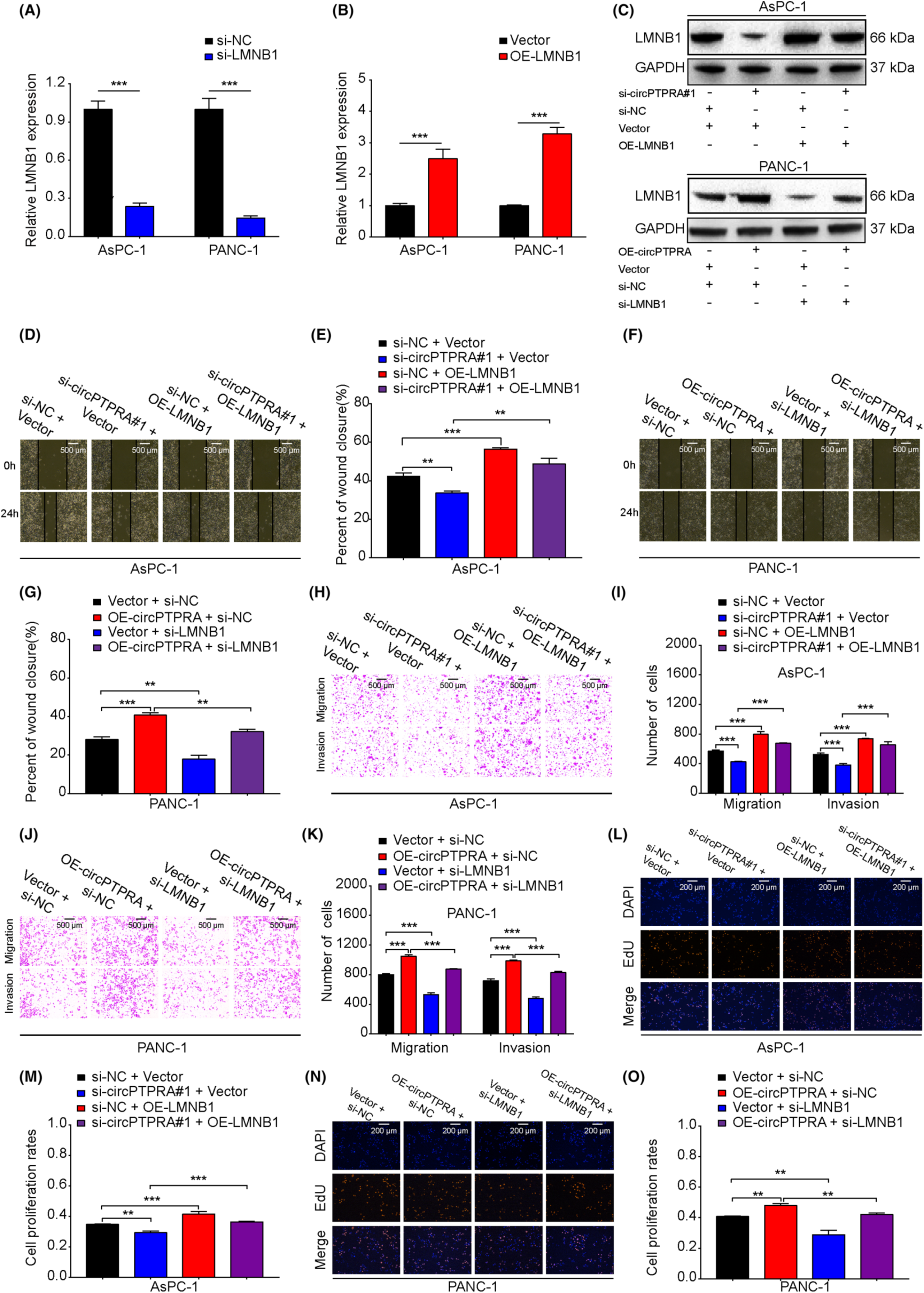
The circPTPRA/miR‐140‐5p/LMNB1 axis promotes PDAC progression in vitro. (A, B) The efficiency of the si‐LMNB1 and OE‐LMNB1 plasmid was verified by qRT–PCR in AsPC‐1 and PANC‐1 cells. Two‐way ANOVA was used. (C) After transfection with si‐circPTPRA#1, OE‐LMNB1 plasmid, si‐circPTPRA#1 + OE‐LMNB1 in AsPC‐1 cells and transfection with OE‐circPTPRA plasmid, si‐LMNB1, OE‐circPTPRA plasmid + si‐LMNB1 in PANC‐1 cells, the protein level of LMNB1 was detected by western blotting. (D–O) AsPC‐1 and PANC‐1 cells were divided into four groups for rescue experiments. The migration, invasion, and proliferation capabilities of circPTPRA and LMNB1 in AsPC‐1 and PANC‐1 cells were evaluated by wound healing (D–G), transwell (H–K) and EdU (L–O) assays. The wound healing and transwell assay images were photographed at 40× magnification. Scale bar = 500 μm, the EdU images were photographed at 200× magnification. Scale bar = 200 μm. One‐way and two‐way ANOVA were used. The above data are presented as the mean ± SD of three independent experiments. *p < 0.05, **p < 0.01, ***p < 0.001.

LMNB1, an oncogene, is also a common downstream target gene of circPTPRA and miR‐140‐5p, but its role in PDAC has not been reported. To investigate whether circPTPRA promoted PDAC progression via the miR‐140‐5p/LMNB1 axis in vitro, we designed and synthesized the siRNA and overexpression plasmid of LMNB1. The efficiency of the siRNA and plasmid was measured by qRT–PCR in AsPC‐1 and PANC‐1 cells (Figure 6A,B). At the protein level, si‐circPTPRA#1 inhibited the expression of LMNB1, but the addition of OE‐LMNB1 could rescue the inhibitory effect of si‐circPTPRA#1 in AsPC‐1 cells. Moreover, OE‐circPTPRA increased the expression of LMNB1, but the above phenotype could be rescued after transfection with si‐LMNB1 in PANC‐1 cells (Figure 6C). Functionally, increased expression of LMNB1 in AsPC‐1 cells promoted cell migration, invasion, proliferation, and reversed the anti‐tumor effects of si‐circPTPRA#1 (Figure 6D,E,H,I,L,M). On the other hand, decreased expression of LMNB1 in PANC‐1 cells significantly inhibited cell migration, invasion, proliferation, and blocked the pro‐tumor effects induced by OE‐circPTPRA (Figure 6F,G,J,K,N,O). Briefly, our results confirmed that LMNB1 promoted PDAC progression in vitro, and it was particularly important that circPTPRA maintained the malignant phenotype of PDAC dependent on the miR‐140‐5p/LMNB1 axis.

### 3.7 The circPTPRA/miR‐140‐5p/LMNB1 axis promotes PDAC progression in vivo

**FIGURE 7:**
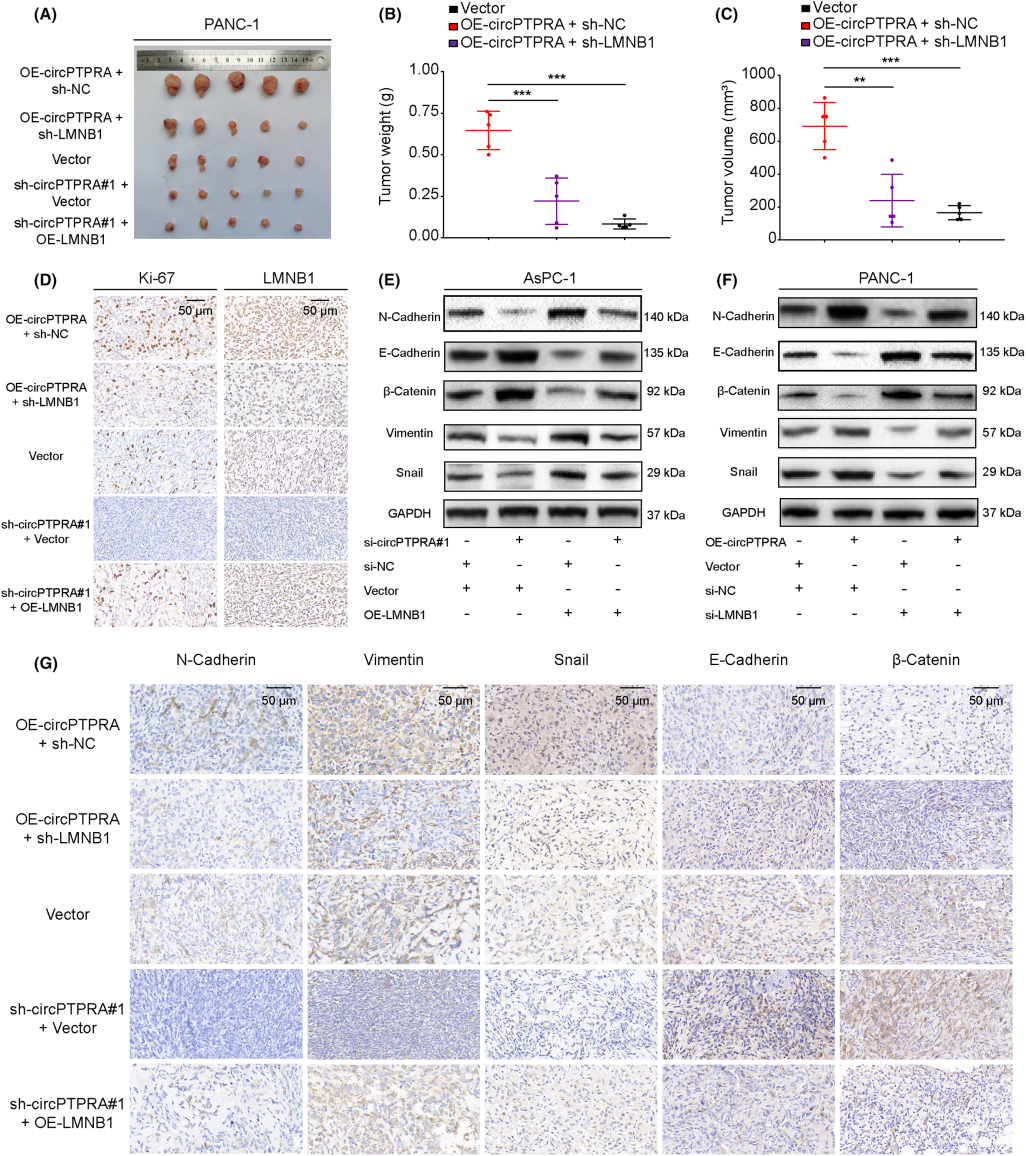
The circPTPRA/miR‐140‐5p/LMNB1 axis promotes PDAC progression in vivo. (A) Representative picture of subcutaneous xenograft tumors (*n* = 5 for each group). (B, C) Tumor weight (g) and tumor volume (mm^3^) were analyzed. One‐way ANOVA was used. (D) IHC staining was used to evaluate Ki‐67 and LMNB1 protein level in subcutaneous xenograft tumor. The images of IHC staining were photographed at 400× magnification. Scale bar = 50 μm. (E, F) After transfection with si‐circPTPRA#1, OE‐LMNB1 plasmid, and si‐circPTPRA#1 + OE‐LMNB1 in AsPC‐1 cells and transfection with OE‐circPTPRA plasmid, si‐LMNB1, and OE‐circPTPRA plasmid + si‐LMNB1 in PANC‐1 cells, the protein level of N‐Cadherin, E‐Cadherin, β‐catenin, Vimentin, and Snail was detected by western blotting. (G) IHC staining was used to evaluate N‐Cadherin, E‐Cadherin, β‐catenin, Vimentin, and Snail protein level in subcutaneous xenograft tumors. The IHC staining images were photographed at 400× magnification. Scale bar = 50 μm.

To further verify the function of the circPTPRA/miR‐140‐5p/LMNB1 axis in PDAC progression in vivo, the subcutaneous tumorigenesis model was established with stably transfected PANC‐1 cells. Nude mice were sacrificed 30 days after inoculation with PANC‐1 cells, and subcutaneous tumor tissues were obtained and photographed (Figure 7A and Additional file 2 Figure S1A). In vivo data confirmed that tumor weight and volume were significantly increased by overexpression of circPTPRA, but sh‐LMNB1 partially rescued those phenotypes (Figure 7B,C). Similarly, tumor weight and volume were significantly reduced by sh‐circPTPRA#1, but OE‐LMNB1 partially reversed the anti‐tumor effects of sh‐circPTPRA#1 (Additional file 2 Figure S1B,C). The IHC results also showed that the expression of LMNB1 and Ki‐67 in the OE‐circPTPRA group was significantly higher than that in the vector group, but sh‐LMNB1 partially rescued those phenotypes. In contrast, the expression level of LMNB1 and ki‐67 was significantly decreased in the sh‐circPTPRA#1 group, but OE‐LMNB1 rescued the above phenotypes (Figure 7D). We further found that interfering with the expression of circPTPRA in AsPC‐1 cells reduced the expression of N‐Cadherin, Vimentin and Snail, and increased the expression of E‐Cadherin and β‐Catenin, but OE‐LMNB1 partially rescued the above phenotypes (Figure 7E). Moreover, overexpression of circPTPRA in PANC‐1 cells increased the expression of N‐Cadherin, Vimentin, Snail and reduced the expression of E‐Cadherin and β‐Catenin, but si‐LMNB1 partially rescued the above phenotypes (Figure 7F). Then, the IHC results were consistent with the results in vitro (Figure 7G). In brief, these data confirmed that the circPTPRA/miR‐140‐5p/LMNB1 axis could promote PDAC progression in vivo.

### 3.8 Overexpression of circPTPRA is closely associated with local lymph node invasion and poor prognosis in PDAC patients

**FIGURE 8:**
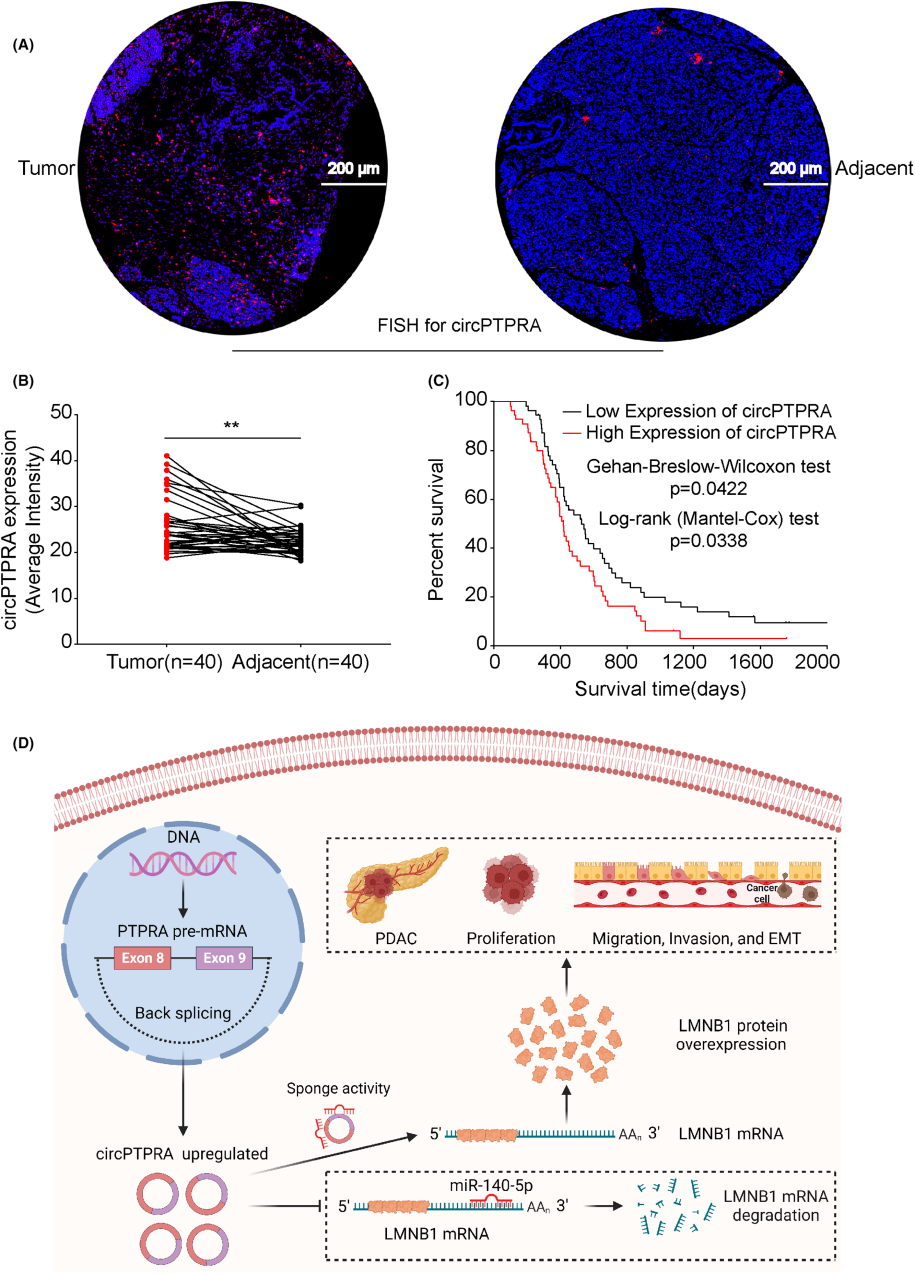
Overexpression of circPTPRA is closely associated with local lymph node invasion and poor prognosis in PDAC patients. (A) Representative FISH picture showing the expression of circPTPRA in PDAC tissues and paired NATs. The FISH images were photographed at 50× magnification. Scale bar = 200 μm. (B) The expression of circPTPRA in PDAC tissues (*n* = 40) and paired NATs (*n* = 40). The Mann–Whitney test was used. (C) Kaplan–Meier survival curves was used to analyze the overall survival of PDAC patients with high versus low circPTPRA expression (the median circPTPRA expression was used as the cut‐off value). (D) Schematic illustration showing the mechanism by which circPTPRA promotes the progression of PDAC via the miR‐140‐5p/LMNB1 axis.

To further evaluate the clinical significance of circPTPRA in PDAC, the FISH assay was used to detect the expression of circPTPRA in tissue microarrays. Table S2 showed the detailed clinical data of 130 PDAC patients (Additional file 3 Table S2). The FISH results confirmed that circPTPRA was significantly overexpressed in PDAC tissues compared with paired NATs (Figure 8A,B). Interestingly, the clinical data of PDAC patients showed that high expression of circPTPRA was closely associated with regional lymph node invasion (Table 1). It is worth noting that compared with patients with low expression of circPTPRA, patients with high expression of circPTPRA had a worse prognosis (Figure 8C).

**TABLE 1:** Correlation of circPTPRA expression with clinical data characteristics of PDAC patients (*n* = 130).

Clinical data characteristics	Total (*n* = 130)	circPTPRA expression		*p* value
		Low (65)	High (65)	
Gender				
Male	82	42	40	
Female	48	23	25	0.5054
Age (years)				
<60	56	29	27	
≥60	74	36	38	0.6784
Grade				
High/moderate	99	47	52	
Low	31	18	13	0.8705
T stage				
T1/T2	73	33	40	
T3/4	16	7	9	0.5011
Data missing	41			
N stage				
N0	89	48	41	
N1/2	41	17	24	0.0125*
M stage				
M0	125	63	62	
M1	5	2	3	0.2639
Nerve invasion				
(−)	94	47	47	
(+)	36	18	18	0.3823
Preoperative serum CA19‐9				
≤40 (U/mL)	17	9	8	
>40 (U/mL)	36	12	24	0.0715
Data missing	77			

## 4 DISCUSSION

With the advancement of high‐throughput sequencing and bioinformatics technology, an increasing number of new circRNAs with important functions have been discovered. We previously measured circRNAs expression in 20 PDAC tissues and paired NATs.^23^ In the present study, we combined our data with the data of GSE69362, and we focused on circRNAs with abnormally high expression in PDAC. We found that circPTPRA was significantly overexpressed in PDAC tissues and cells. The results of gain‐ and loss‐of‐function experiments demonstrated that circPTPRA promotes the migration, invasion, proliferation and EMT of PDAC cells in vitro and in vivo, suggesting the pro‐tumor role of circPTPRA in PDAC. However, several studies have reported that circPTPRA upregulates KLF9 by sponging miR‐636 or interacting with IGF2BP1 to inhibit the progression of bladder cancer.^32^, ^33^ Thus, circPTPRA can play opposite roles in different tumors, which may be related to tumor heterogeneity. Studies have reported that miR‐636 acts as a tumor suppressor^34^ or oncogene^33^ in other tumors, but its role in PDAC is unclear. IGF2BP1 promotes PDAC cell proliferation by activating AKT, which can be used as a driver and a therapeutic target in PDAC.^35^ It is worth considering whether there are synergistic effects and regulatory mechanisms between circPTPRA and IGF2BP1 in PDAC, which would be a meaningful research direction. The above studies confirmed the key role of circPTPRA in tumors and indicated that circPTPRA has significant research value.

It has been reported that the regulation of downstream gene expression by sponging miRNAs is one of the main functions of circRNAs.^18^ The circPTPRA‐miRNAs interaction was predicted with the TargetScan and miRanda.^27^, ^28^ We found that five miRNAs (miR‐140‐5p, miR‐152‐5p, miR‐582‐3p, miR‐26b‐3p, miR‐96‐5p) may directly bind to circPTPRA. Among them, only miR‐140‐5p was significantly enriched by the biotin‐labeled circPTPRA probe in both AsPC‐1 and PANC‐1 cells. The results in Figure 3D,E also showed that miR‐96‐5p could be enriched by circPTPRA probe in AsPC‐1 cells and miR‐582‐3p could be enriched by the circPTPRA probe in PANC‐1 cells, which may be caused by cell type specificity. This similar phenomenon has also been confirmed by other articles.^36^, ^37^ To screen for a universal miRNA target for circPTPRA in AsPC‐1 and PANC‐1 cells, we selected miR140‐5p for further validation. Studies have revealed the tumor suppressor function of miR‐140‐5p in digestive system malignancies. Hao Yang et al. reported that miR‐140‐5p inhibits the growth and metastasis of hepatocellular carcinoma by targeting TGFBR1 and FGF9.^38^ Zheng Fang et al. confirmed that miR‐140‐5p regulates the downstream target gene YES1 to inhibit the proliferation, migration, and invasion of gastric cancer.^39^ Bo Yuan et al. revealed that the miR‐140‐5p/CDK8 axis is regulated by lncRNA HCP5 to promote PDAC progression.^30^ In our study, LMNB1 was identified as a common downstream target gene of circPTPRA and miR‐140‐5p by whole‐transcriptome sequencing and bioinformatics analysis. Dual‐luciferase reporter assay results showed that miR‐140‐5p directly binds to the “AACCACT” base sequence in the 3′UTR of LMNB1. Moreover, the IHC results showed that LMNB1 was highly expressed in PDAC, positively correlated with the expression of circPTPRA, and negatively correlated with the expression of miR‐140‐5p. In addition, LMNB1 and circPTPRA synergistically promoted PDAC migration, invasion, proliferation, and EMT. Overall, our study confirmed that circPTPRA can mediate PDAC progression through the circPTPRA/140‐5p/LMNB1 axis.

LMNB1 is a member of the lamins family,^40^ which is necessary for cell survival and plays an important role in the regulation of cell proliferation and senescence, chromosome distribution condensation, and cellular DNA damage repair.^41^, ^42^, ^43^, ^44^, ^45^ In addition, LMNB1 also plays an important role in tumor progression, and the biological function of LMNB1 is closely related to the abnormal proliferation and potential tumorigenicity of tumor cells. For example, LMNB1facilitates cell proliferation, EMT, and metastasis in hepatocellular carcinoma,^46^ LMNB1 promotes lung adenocarcinoma cell proliferation through the AKT pathway.^47^ Most importantly, LMNB1 is a potential therapeutic target for betulinic acid in PDAC,^48^ but the molecular mechanism by which LMNB1 promotes PDAC progression is unclear. In this paper, we revealed that the overexpression of circPTPRA is closely related to local lymph node infiltration in PDAC patients and we first confirmed that circPTPRA/140‐5p/LMNB1 axis promotes PDAC migration, invasion, proliferation, and EMT in vivo and in vitro. It is worth noting that we only detected the relevant biomarkers of EMT to confirm that circPTPRA/140‐5p/LMNB1 axis is related to the EMT process of PDAC. Considering that EMT is a complex process involving multiple genes and signal pathways,^49^ it will be a very promising research topic to analyze the function of circPTPRA/miR‐140‐5p/LMNB1 axis in different stages of EMT.

As everyone knows, the regulation of circRNAs formation depends on multiple factors^50^ and RNA binding proteins play the most critical role in the generation of circRNAs. For instance, EIF4A3 can induce the expression of circMMP9 and circASAP1,^51^, ^52^ FUS can induce abnormally high expression of circRHOBTB3 and circ_002136 in tumors.^53^, ^54^ Through analysis of the Circular RNA Interactome online database,^55^ we found that EIF4A3 and FUS contain binding sites for circPTPRA. To verify whether EIF4A3 and FUS could promote circPTPRA formation, we constructed overexpression plasmids and siRNAs for EIF4A3 and FUS, and western blotting was used to detect the efficiency of the plasmids and siRNAs (Additional file 4 Figure S2A,D). Unfortunately, the qRT–PCR results showed that neither gain‐ nor loss‐of‐function of EIF4A3 or FUS could affect the expression of circPTPRA (Additional file 4 Figure S2B,C,E,F). The synthesis of circRNAs is regulated by multiple pathways which contribute to the diverse expression of circRNAs in different cell types and under different disease conditions. We speculate that circPTPRA is upregulated by other processes in PDAC, including precursor RNA transcription and post‐or co‐transcriptional processing and turnover. Therefore, more efforts are needed to uncover why circPTPRA is abnormally upregulated in PDAC, which would be a meaningful study.

There are still deficiencies in this study. First, circPTPRA plays an important role in tumor progression. In this study, we only revealed its ceRNA mechanism in PDAC, which does not mean that there are no other important regulatory networks, so mechanisms in other areas need further exploration. Second, LMNB1 is a potential therapeutic target of betulinic acid in PDAC. Our study has not confirmed whether si‐circPTPRA can promote the therapeutic effect of betulinic acid on PDAC, which will be our next research plan. Third, the synthesis and degradation of circRNAs are complex processes. In this study, we failed to elucidate the reason for the abnormally high expression of circPTPRA in PDAC, which requires more work to explain. Finally, our results show that circPTPRA can promote the proliferation of PDAC cells in vitro and in vivo, but there was no significant correlation between circPTPRA expression and patient T stage. The reason could be that some patients' data were missing, and a small number of cases are not enough to obtain meaningful analysis results. The results of this study need to be validated in multicenter studies.

In conclusion, our study confirmed that circPTPRA was abnormally upregulated in PDAC and closely associated with local lymph node invasion and poor prognosis. More importantly, the activation of the circPTPRA/miR‐140‐5p/LMNB1 signaling pathway promoted PDAC migration, invasion, proliferation, and EMT in vitro and in vivo (Figure 8D). However, the mechanism of circPTPRA activation needs further study, and the expression of circPTPRA should be further evaluated in blood, urine, and exosomes to improve its clinical application value.

## AUTHOR CONTRIBUTIONS

**Wen Fu:** Data curation (lead); investigation (lead); resources (lead); writing – original draft (lead); writing – review and editing (lead). **Xianxing Wang:** Formal analysis (supporting); investigation (supporting); writing – review and editing (supporting). **Jifeng Xiang:** Data curation (supporting); formal analysis (supporting). **Shengkai Chen:** Data curation (supporting); formal analysis (supporting). **Renpai Xia:** Investigation (supporting); resources (supporting). **Fuming Xie:** Investigation (supporting); resources (supporting). **Bojing Chi:** Investigation (supporting); resources (supporting). **Fanbo Qin:** Investigation (supporting); resources (supporting). **Zhuo Li:** Investigation (supporting); resources (supporting). **Li Mou:** Conceptualization (equal); methodology (supporting); resources (supporting); writing – review and editing (supporting). **Chuanming Xie:** Conceptualization (supporting); methodology (equal); resources (supporting); writing – review and editing (supporting). **Huaizhi Wang:** Conceptualization (equal); methodology (equal); resources (equal); writing – review and editing (equal).

## ETHICAL APPROVAL STATEMENT

Informed consent was obtained from all patients prior to obtaining tissue samples, and all procedures were approved by the Medical Ethics Committee of Chongqing People's Hospital (KY 2021–039‐01).

## Supporting information


Data S1

## Data Availability

This microarray data was deposited in the NCBI Gene Expression Omnibus (GEO) datasets under the accession number GSE79634 and GSE69362. The authors declare that all the data supporting the findings in this study are available in this study and supplementary information.
