# Supplementary material for: CircPTPRA promotes the progression of pancreatic ductal adenocarcinoma via the miR‐140‐5p/LMNB1 axis
Source: Cancer Med. 2023 Apr 11;12(10):11651–71. doi: 10.1002/cam4.5869 (PMC10242365; doi:10.1002/cam4.5869)
Supplement: Supplementary file 1 — Data S1 [file CAM4-12-11651-s001.docx]

**TableS1** The sequences used in this article are shown below

| Gene name | Sequence (5'-3') | Application |
| --- | --- | --- |
| circPTPRA | F: GAAACTTTCCCTCCTTCAGAT | qRT-PCR |
|  | R: CTACAGAAGGTGCAACTGTG |  |
| PTPRA | F: TGGATGATGCAGTTCAAATAACTAA | qRT-PCR |
|  | R: GGTGCTTGCTGTTCTTGTGG |  |
| EIF4A3 | F: GCTGCTTGCTCTCGGTGACTAC | qRT-PCR |
|  | R: GCTTCCTGATGTCCTCGCCAAC |  |
| FUS | F: GGTACTCAGCGGTGTTGGAA | qRT-PCR |
|  | R: GTTCTGGCTCTGGCCATAAGA |  |
| LMNB1 | F: GCGGTGTACATCGACAAGGTG | qRT-PCR |
|  | R: TTGCACTTGCCCAGCTCGAT |  |
| GAPDH | F: GGCAAATTCCATGGCACCGT | qRT-PCR |
|  | R: TGGACTCCACGACGTACTCA |  |
| miR-140-5p mimics | CAGUGGUUUUACCCUAUGGUAG | mimics |
| miR-140-5p mimics NC | UCACAACCUCCUAGAAAGAGUAGA |  |
| miR-140-5p inhibitor | CTACCATAGGGTAAAACCACTG | inhibitor |
| miR-140-5p inhibitor NC | UUUGUACUACACAAAAGUACUG |  |
| si-circPTPRA#1 | CCTCCTTCAGATAAGCATG | siRNA |
| si-circPTPRA#2 | TTCAGATAAGCATGGATTC | siRNA |
| si-LMNB1 | CGAGCATCCTCAAGTCGTA | siRNA |
| si-EIF4A3 | CGAGCAATCAAGCAGATCA | siRNA |
| si-FUS | CAAGCAGATTGGTATTATT | siRNA |
| si-NC | GGCUCUAGAAAAGCCUAUGCdTdT | siRNA |
| FAM-labeled miR-140-5p probe | CTACCATAGGGTAAAACCACTG | FISH |
| Cy3-labeled circPTPRA probe 1 | ATGCTTATCTGAAGGAGGGAAAGTT | FISH |
| Cy3-labeled circPTPRA probe 2 | TCCATGCTTATCTGAAGGAGGGAAA | FISH |
| Biotin-labeled circPTPRA probe | TGCTTATCTGAAGGAGGGAA | RNA pull down |
| circPTPRA overexpression vector | TCGGTCCGATTTAAATTGAAAAAAATTGTTGACATTAATATTTCTTCTTTCGAATTCTAATACTTTCAGGTGCCTTAGCTGGACCAATTATTGTGGAGCCACATGTCACAGCAGTATGGGGAAAGAATGTTTCATTAAAGTGTTTAATTGAAGTAAATGAAACCATAACACAGATTTCATGGGAGAAGATACATGGCAAAAGTTCACAGACTGTTGCAGTTCACCATCCCCAATATGGATTCTCTGTTCAAGGAGAATATCAGGGAAGAGTCTTGTTTAAAAATTACTCACTTAATGATGCAACAATTACTCTGCATAACATAGGATTCTCTGATTCTGGAAAATACATCTGCAAAGCTGTTACATTCCCGCTTGGAAATGCCCAGTCCTCTACAACTGTAACTGTGTTAGTTGAACCCACTGTGAGCCTGATAAAAGGGCCAGATTCTTTAATTGATGGAGGAAATGAAACAGTAGCAGCCATTTGCATCGCAGCCACTGGAAAACCCGTTGCACATATTGACTGGGAAGGTGATCTTGGTGAAATGGAATCCACTACAACTTCTTTTCCAAATGAAACGGCAACGATTATCAGCCAGTACAAGCTATTTCCAACCAGATTTGCTAGAGGAAGGCGAATTACTTGTGTTGTAAAACATCCAGCCTTGGAAAAGGACATCCGATACTCTTTCATATTAGACATACAGTATGCTCCTGAAGTTTCGGTAACAGGATATGATGGAAATTGGTTTGTAGGAAGAAAAGGTGTTAATCTCAAATGTAATGCTGATGCAAATCCACCACCCTTCAAATCTGTGTGGAGCAGTAAGAACAACTGGATCCTAGCTAACAACTCCATACTTTTTGGTTGTTTATTAATGTGAAATTTCTGCTAAATGAAATACTTTTGTGTGTGTTTGTGGTAGAAGAGACCACTTCAGTTAAATAAGGAAATCAAGAGAGGATCAATTTAGAAGATTCAGATATACAGCCCGGGTGCAGTGGCTCATGCCTGTAATCCCTGCACTTAAGGGAGCTGAGGCGGGTGGATGACTGAGGTTAGGAGTTTCAGACCAGCCTGCAACATGCGAAACCCCATCTCTACTAAATAACAAATAGCTGATGTGTGTTGCTGTTCTATATCCCAGCACTGGAGCTTAAGCCAGGAATTCACGAAGAATCTGCGATTCGCCTCCGATCTCCGTTCAATGGGCCAGAGAGCTGCCACAATCGG | Plasmid |
| EIF4A3 overexpression vector | ATGGCGACCACGGCCACGATGGCGACCTCGGGCTCGGCGCGAAAGCGGCTGCTCAAAGAGGAAGACATGACTAAAGTGGAATTCGAGACCAGCGAGGAGGTGGATGTGACCCCCACGTTCGACACCATGGGCCTGCGGGAGGACCTGCTGCGGGGCATCTACGCTTACGGTTTTGAAAAACCATCAGCAATCCAGCAACGAGCAATCAAGCAGATCATCAAAGGGAGAGATGTCATCGCACAGTCTCAGTCCGGCACAGGAAAAACAGCCACCTTCAGTATCTCAGTCCTCCAGTGTTTGGATATTCAGGTTCGTGAAACTCAAGCTTTGATCTTGGCTCCCACAAGAGAGTTGGCTGTGCAGATCCAGAAGGGGCTGCTTGCTCTCGGTGACTACATGAATGTCCAGTGCCATGCCTGCATTGGAGGCACCAATGTTGGCGAGGACATCAGGAAGCTGGATTACGGACAGCATGTTGTCGCGGGCACTCCAGGGCGTGTTTTTGATATGATTCGTCGCAGAAGCCTAAGGACACGTGCTATCAAAATGTTGGTTTTGGATGAAGCTGATGAAATGTTGAATAAAGGTTTCAAAGAGCAGATTTACGATGTATACAGGTACCTGCCTCCAGCCACACAGGTGGTTCTCATCAGTGCCACGCTGCCACACGAGATTCTGGAGATGACCAACAAGTTCATGACCGACCCAATCCGCATCTTGGTGAAACGTGATGAATTGACTCTGGAAGGCATCAAGCAATTTTTCGTGGCAGTGGAGAGGGAAGAGTGGAAATTTGACACTCTGTGTGACCTCTACGACACACTGACCATCACTCAGGCGGTCATCTTCTGCAACACCAAAAGAAAGGTGGACTGGCTGACGGAGAAAATGAGGGAAGCCAACTTCACTGTATCCTCAATGCATGGAGACATGCCCCAGAAAGAGCGGGAGTCCATCATGAAGGAGTTCCGGTCGGGCGCCAGCCGAGTGCTTATTTCTACAGATGTCTGGGCCAGGGGGTTGGATGTCCCTCAGGTGTCCCTCATCATTAACTATGATCTCCCTAATAACAGAGAATTGTACATACACAGAATTGGGAGATCAGGTCGATACGGCCGGAAGGGTGTGGCCATTAACTTTGTAAAGAATGACGACATCCGCATCCTCAGAGATATCGAGCAGTACTATTCCACTCAGATTGATGAGATGCCGATGAACGTTGCTGATCTTATCTGA | Plasmid |
| FUS overexpression vector | ATGGCCTCAAACGATTATACCCAACAAGCAACCCAAAGCTATGGGGCCTACCCCACCCAGCCCGGGCAGGGCTATTCCCAGCAGAGCAGTCAGCCCTACGGACAGCAGAGTTACAGTGGTTATAGCCAGTCCACGGACACTTCAGGCTATGGCCAGAGCAGCTATTCTTCTTATGGCCAGAGCCAGAACACAGGCTATGGAACTCAGTCAACTCCCCAGGGATATGGCTCGACTGGCGGCTATGGCAGTAGCCAGAGCTCCCAATCGTCTTACGGGCAGCAGTCCTCCTACCCTGGCTATGGCCAGCAGCCAGCTCCCAGCAGCACCTCGGGAAGTTACGGTAGCAGTTCTCAGAGCAGCAGCTATGGGCAGCCCCAGAGTGGGAGCTACAGCCAGCAGCCTAGCTATGGTGGACAGCAGCAAAGCTATGGACAGCAGCAAAGCTATAATCCCCCTCAGGGCTATGGACAGCAGAACCAGTACAACAGCAGCAGTGGTGGTGGAGGTGGAGGTGGAGGTGGAGGTAACTATGGCCAAGATCAATCCTCCATGAGTAGTGGTGGTGGCAGTGGTGGCGGTTATGGCAATCAAGACCAGAGTGGTGGAGGTGGCAGCGGTGGCTATGGACAGCAGGACCGTGGAGGCCGCGGCAGGGGTGGCAGTGGTGGCGGCGGCGGCGGCGGCGGTGGTGGTTACAACCGCAGCAGTGGTGGCTATGAACCCAGAGGTCGTGGAGGTGGCCGTGGAGGCAGAGGTGGCATGGGCGGAAGTGACCGTGGTGGCTTCAATAAATTTGGTGGCCCTCGGGACCAAGGATCACGTCATGACTCCGAACAGGATAATTCAGACAACAACACCATCTTTGTGCAAGGCCTGGGTGAGAATGTTACAATTGAGTCTGTGGCTGATTACTTCAAGCAGATTGGTATTATTAAGACAAACAAGAAAACGGGACAGCCCATGATTAATTTGTACACAGACAGGGAAACTGGCAAGCTGAAGGGAGAGGCAACGGTCTCTTTTGATGACCCACCTTCAGCTAAAGCAGCTATTGACTGGTTTGATGGTAAAGAATTCTCCGGAAATCCTATCAAGGTCTCATTTGCTACTCGCCGGGCAGACTTTAATCGGGGTGGTGGCAATGGTCGTGGAGGCCGAGGGCGAGGAGGACCCATGGGCCGTGGAGGCTATGGAGGTGGTGGCAGTGGTGGTGGTGGCCGAGGAGGATTTCCCAGTGGAGGTGGTGGCGGTGGAGGACAGCAGCGAGCTGGTGACTGGAAGTGTCCTAATCCCACCTGTGAGAATATGAACTTCTCTTGGAGGAATGAATGCAACCAGTGTAAGGCCCCTAAACCAGATGGCCCAGGAGGGGGACCAGGTGGCTCTCACATGGGGGGTAACTACGGGGATGATCGTCGTGGTGGCAGAGGAGGCTATGATCGAGGCGGCTACCGGGGCCGCGGCGGGGACCGTGGAGGCTTCCGAGGGGGCCGGGGTGGTGGGGACAGAGGTGGCTTTGGCCCTGGCAAGATGGATTCCAGGGGTGAGCACAGACAGGATCGCAGGGAGAGGCCGTATTAA | Plasmid |
| LMNB1 overexpression vector | CTGGCCGTTTTTGGCTTTTTTGTTAGACGAAGCTTGGGCTGCAGGTCGACTCTAGAGGATCCCGCCACCATGGCGACTGCGACCCCCGTGCCGCCGCGGATGGGCAGCCGCGCTGGCGGCCCCACCACGCCGCTGAGCCCCACGCGCCTGTCGCGGCTCCAGGAGAAGGAGGAGCTGCGCGAGCTCAATGACCGGCTGGCGGTGTACATCGACAAGGTGCGCAGCCTGGAGACGGAGAACAGCGCGCTGCAGCTGCAGGTGACGGAGCGCGAGGAGGTGCGCGGCCGTGAGCTCACCGGCCTCAAGGCGCTCTACGAGACCGAGCTGGCCGACGCGCGACGCGCGCTCGACGACACGGCCCGCGAGCGCGCCAAGCTGCAGATCGAGCTGGGCAAGTGCAAGGCGGAACACGACCAGCTGCTCCTCAACTATGCTAAGAAGGAATCTGATCTTAATGGCGCCCAGATCAAGCTTCGAGAATATGAAGCAGCACTGAATTCGAAAGATGCAGCTCTTGCTACTGCACTTGGTGACAAAAAAAGTTTAGAGGGAGATTTGGAGGATCTGAAGGATCAGATTGCCCAGTTGGAAGCCTCCTTAGCTGCAGCCAAAAAACAGTTAGCAGATGAAACTTTACTTAAAGTAGATTTGGAGAATCGTTGTCAGAGCCTTACTGAGGACTTGGAGTTTCGCAAAAGCATGTATGAAGAGGAGATTAACGAGACCAGAAGGAAGCATGAAACGCGCTTGGTAGAGGTGGATTCTGGGCGTCAAATTGAGTATGAGTACAAGCTGGCGCAAGCCCTTCATGAGATGAGAGAGCAACATGATGCCCAAGTGAGGCTGTATAAGGAGGAGCTGGAGCAGACTTACCATGCCAAACTTGAGAATGCCAGACTGTCATCAGAGATGAATACTTCTACTGTCAACAGTGCCAGGGAAGAACTGATGGAAAGCCGCATGAGAATTGAGAGCCTTTCATCCCAGCTTTCTAATCTACAGAAAGAGTCTAGAGCATGTTTGGAAAGGATTCAAGAATTAGAGGACTTGCTTGCTAAAGAAAAAGACAACTCTCGTCGCATGCTGACAGACAAAGAGAGAGAGATGGCGGAAATAAGGGATCAAATGCAGCAACAGCTGAATGACTATGAACAGCTTCTTGATGTAAAGTTAGCCCTGGACATGGAAATCAGTGCTTACAGGAAACTCTTAGAAGGCGAAGAAGAGAGGTTGAAGCTGTCTCCAAGCCCTTCTTCCCGTGTGACAGTATCCCGAGCATCCTCAAGTCGTAGTGTACGTACAACTAGAGGAAAGCGGAAGAGGGTTGATGTGGAAGAATCAGAGGCGAGTAGTAGTGTTAGCATCTCTCATTCCGCCTCAGCCACTGGAAATGTTTGCATCGAAGAAATTGATGTTGATGGGAAATTTATCCGCTTGAAGAACACTTCTGAACAGGATCAACCAATGGGAGGCTGGGAGATGATCAGAAAAATTGGAGACACATCAGTCAGTTATAAATATACCTCAAGATATGTGCTGAAGGCAGGCCAGACTGTTACAATTTGGGCTGCAAACGCTGGTGTCACAGCCAGCCCCCCAACTGACCTCATCTGGAAGAACCAGAACTCGTGGGGCACTGGCGAAGATGTGAAGGTTATATTGAAAAATTCTCAGGGAGAGGAGGTTGCTCAAAGAAGTACAGTCTTTAAAACAACCATACCTGAAGAAGAGGAGGAGGAGGAAGAAGCAGCTGGAGTGGTTGTTGAGGAAGAACTTTTCCACCAGCAGGGAACCCCAAGAGCATCCAATAGAAGCTGTGCAATTATGTAAGCTAGCACATAACTTACGGTAAATGGCCCGCCTGGCTGACCGCCCAACGACCCCCGCCCATTGACGTCAATAGTAACGCCAATAGGGACTTTCCATTGACGTCAATGGGTGGAGTATTTACGGTAAACTGCCCACTTGGCAGTACATCAAGTGTATCATATGCCAAGTACGCCCCCTATTGACGTCAATGACGGTAAATGGCCCGCCTGGCATTGTGCCCAG | Plasmid |
| sh-circPTPRA#1 | CAGCATCCAACTAAAGATTACAAAAACAAATTACAAAAATTCAAAATTTTCGGGTTTATTACAGGGACAGCAGAGATCCAGTTTGGTTAATTAATCGAGCGGCCGCCCCCTTCACCGAGGGCCTATTTCCCATGATTCCTTCATATTTGCATATACGATACAAGGCTGTTAGAGAGATAATTGGAATTAATTTGACTGTAAACACAAAGATATTAGTACAAAATACGTGACGTAGAAAGTAATAATTTCTTGGGTAGTTTGCAGTTTTAAAATTATGTTTTAAAATGGACTATCATATGCTTACCGTAACTTGAAAGTATTTCGATTTCTTGGCTTTATATATCTTGTGGAAAGGACGAAACACCGGCCTCCTTCAGATAAGCATGCTCGAGCATGCTTATCTGAAGGAGGTTTTTGAATTCTCGACCTCGAGACAAATGGCAGTATTCATCCACGGATCCTAACCCGTGTCGGCTCCAACATAACTTACGGTAAATGGCCCGCCTGGCTGACCGCCCAACGACCCCCGCCCATTGACGTCAATAGTAACGCCAATAGGGACTTTCCATTGACGTCAATGGGTGGAGTATTTACGGTAAACTGCCCACTTGGCAGTACATCAAGTGTATCATATGCCAAGTACGCCCCCTATTGACGTCAATGACGGTAAATGGCCCGCCTGGCATTGTGCCCAGTACATGACCTTATGGGACTTTCCTACTTGGCAGTACATCTACGTATTAGTCATCGCTATTACCATGGTCGAGGTGAGCCCCACGTTCTGCTTCACTCTCCCCATCTCCCCCCCCTCCCCACCCCCAATTTTGTATTTATTTATTTTTTAATTATTTTGTGCAGCGATGGGGGCGGGGGGGGGGGGGGGGGGCG | sh-RNA |
| sh-LMNB1 | GAGACCCTCAACTAAAGATTACAAAACAAATTACAAAAATTCAAAATTTTCGGGTTTATTACAGGGACAGCAGAGATCCAGTTTGGTTAATTAATCGAGCGGCCGCCCCCTTCACCGAGGGCCTATTTCCCATGATTCCTTCATATTTGCATATACGATACAAGGCTGTTAGAGAGATAATTGGAATTAATTTGACTGTAAACACAAAGATATTAGTACAAAATACGTGACGTAGAAAGTAATAATTTCTTGGGTAGTTTGCAGTTTTAAAATTATGTTTTAAAATGGACTATCATATGCTTACCGTAACTTGAAAGTATTTCGATTTCTTGGCTTTATATATCTTGTGGAAAGGACGAAACACCGGCGAGCATCCTCAAGTCGTACTCGAGGAATCCATGCTTATCTGAATTTTTGGAATTCTCGACCTCGAGACAAATGGCAGTATTCATCCACGGATCCTAACCCGTGTCGGCTCCAACATAACTTACGGTAAATGGCCCGCCTGGCTGACCGCCCAACGACCCCCGCCCATTGACGTCAATAGTAACGCCAATAGGGACTTTCCATTGACGTCAATGGGTGGAGTATTTACGGTAAACTGCCCACTTGGCAGTACATCAAGTGTATCATATGCCAAGTACGCCCCCTATTGACGTCAATGACGGTAAATGGCCCGCCTGGCATTGTGCCCAGTACATGACCTTATGGGACTTTCCTACTTGGCAGTACATCTACGTATTAGTCATCGCTATTACCATGGTCGAGGTGAGCCCCACGTTCTGCTTCACTCTCCCCATCTCCCCCCCCTCCCCACCCCCAATTTTGTATTTATTTATTTTTTAATTATTTTGTGCAGCGATGGGGGCGGGGGGGGGGGGGGGGGCGCGCGCCAGGCGGGGCGGGGCGGGGCGAGGGGCGGGGCGGGGCGAGGCGAAAAGGTGCGGCGGCAGCCAATCAAAGCGGCGCGCTCCAAAGTTCCTTTTATATGCGAGCCGCGC | sh-RNA |


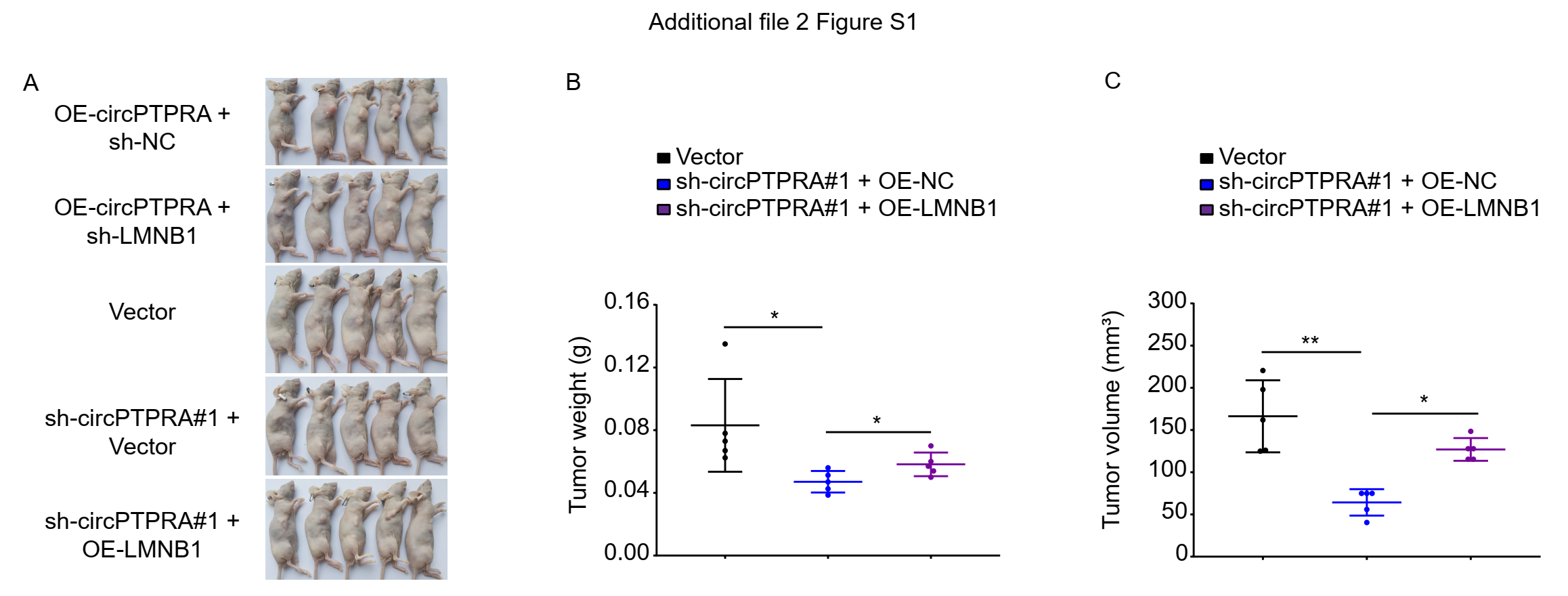


**Additional file 2 Fig. S1. A.** Representative images of nude mouse subcutaneous tumor model (n = 5 for each group). **B-C.** Tumor weight (g) and tumor volume (mm^3^) were analyzed. One-way ANOVA was used.

**Additional file 3 Table S2** The detailed clinical data of 130 PDAC patients

| \| Clinical indicators \| Cases \| \| --- \| --- \| |
| --- | --- | --- |
| \| Gender \|  \| \| --- \| --- \| \| Male \| 82 \| \| Female \| 48 \| \| Age (years) \|  \| \| < 60 \| 56 \| \| ≥ 60 \| 74 \| \| Tumor size (cm) \|  \| \| ≤ 4 \| 73 \| \| > 4 \| 16 \| \| Data missing \| 41 \| \| Lymph node invasion \|  \| \| (-) \| 89 \| \| (+) \| 41 \| \| Nerve invasion \|  \| \| (-) \| 94 \| \| (+) \| 36 \| \| Preoperative serum CA19-9 \|  \| \| ≤ 40 (U/ml) \| 17 \| \| > 40 (U/ml) \| 36 \| \| Data missing \| 77 \| \| Grade \|  \| \| High \| 6 \| \| Moderate \| 93 \| \| Low \| 31 \| |


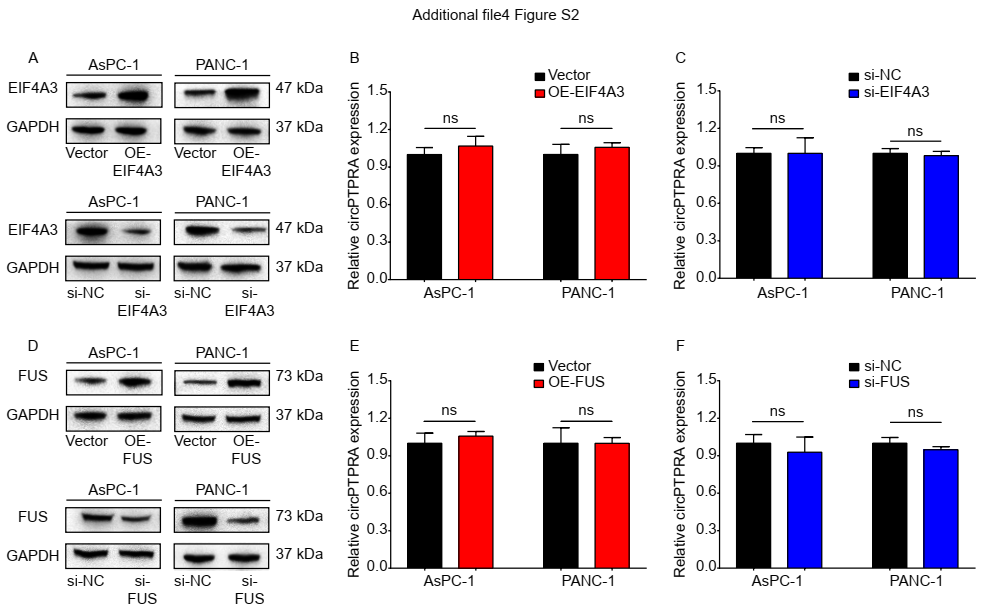


**Additional file 4 Fig. S2. A-C.** After OE-EIF4A3 plasmid and si-EIF4A3 transfection in AsPC-1 and PANC-1 cells, the protein level of EIF4A3 was detected by western blotting, and the relative expression level of circPTPRA was detected by qRT‒PCR. **D-F.** After OE-FUS plasmid and si-FUS transfection in AsPC-1 and PANC-1 cells, the protein level of FUS was detected by western blotting, and the relative expression level of circPTPRA was detected by qRT‒PCR. Two-way ANOVA was used. The above data are presented as the mean ± SD of three independent experiments. **p* < 0.05, ***p* < 0.01, ****p* < 0.001.
